# A New Class of BRCA1 Mimetics for ERα-Positive Breast Cancer Therapy: Design, Synthesis, In Silico Screening, In Vitro Assay, and Gene Expression Analysis

**DOI:** 10.3390/life15040581

**Published:** 2025-04-01

**Authors:** Pottabathula Shyam Sundar, Jubie Selvaraj, Veerachamy Alagarsamy, Viswas Raja Solomon, Jawahar Natarajan

**Affiliations:** 1 Department of Pharmaceutical Chemistry, Vasantidevi Patil Institute of Pharmacy, Kodoli 416114, Maharastra, India; shyam049@gmail.com; 2 Department of Pharmaceutical Chemistry, JSS College of Pharmacy, JSS Academy of Higher Education & Research, Ooty 643001, Tamilnadu, India; 3 Department of Pharmaceutical Chemistry, MNR College of Pharmacy, Sangareddy 502294, Telangana, India; profvalagarsamy@gmail.com; 4 Department of Chemistry, University of Saskatchewan, Saskatoon, SK S7N 5A2, Canada; 5 Department of Pharmaceutics, JSS College of Pharmacy, JSS Academy of Higher Education & Research, Ooty 643001, Tamilnadu, India; jawahar.n@jssuni.edu.in

**Keywords:** BRCA1, cyclin D1, BCL2, Glide XP, MMGBSA, qRT-PCR, ERα, breast cancer, gene expression

## Abstract

Breast Cancer Gene 1 (BRCA1) offers a potential approach for ERα repression by blocking cyclin D1’s interaction with ERα, which prevents cells from growing and dividing too rapidly or uncontrollably. When BRCA1 levels are low, BRCA1 mimetics fit into the BRCA1-binding pocket within ERα, mimicking the ability of BRCA1 to inhibit ERα activity. This study aims to identify a novel class of lead molecules for BRCA1 mimetics for ER-positive breast cancer, distinct from conventional antiestrogen therapies in their mechanism of action. In this article, coumarin thiosemicarbazone hybrids were synthesized from 7-hydroxy 4-methyl coumarin/4-hydroxy coumarin and thiosemicarbazide with different aldehydes and evaluated for their ERα repression activity. The most active compounds in the series, **9b**, **9l**, and **9m**, exhibited significant potency with an IC_50_ value of 14.49 µM, 35.08 µM and 42.12 µM, respectively, compared to raloxifene (reported) as the positive control with an IC_50_ value of 13.7 µM. The gene expression study confirmed the downregulation of the cyclin D1 gene for the compounds **9l** (−0.217) and **9m** (−0.214). Similarly, the downregulation of the BCL2 gene for the compounds **9b** (−0.373), **9l** (−0.320), and **9m** (−0.376). Also, molecular docking studies and MMGBSA were performed to determine key interactions between compounds and ERα at the BRCA1 binding pocket (AA 338–387). In silico, ADMET properties were executed to illustrate the druggability and safety of the novel derivatives. In silico, in vitro, and gene expression studies revealed that among all the compounds, **9b**, **9l**, and **9m** are promising candidates for the development of lead molecules targeting ERα inhibitors for breast cancer treatment. Moreover, the concept of ERα repression with small molecules as BRCA1 mimetics is novel. In general, it can be concluded that these compounds can serve as promising leads to the design of potential BRCA1 mimetics.

## 1. Introduction

**Figure 1:**
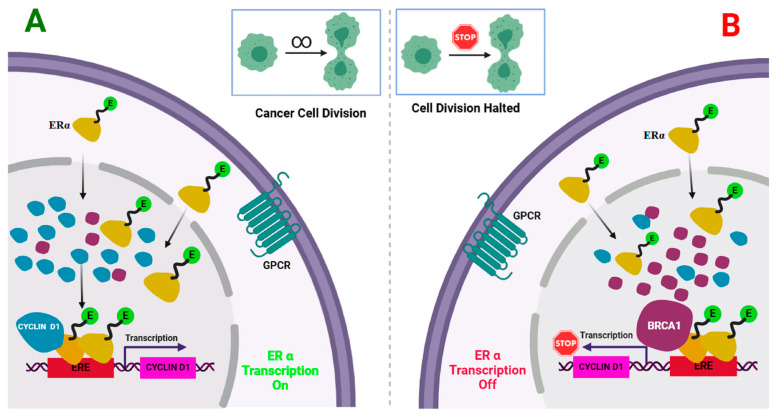
(Created with BioRender.com). (**A**) In the case when BRCA1 is mutated, or there is a lack of BRCA1 in the breast cancer cell, 17-β estradiol liganded ERα enters into the nucleus, undergoes dimerization, binds with ERE (Estrogen Receptor Elements), and recruits CyclinD1 as co-activator forms in the transcription complex. This complex initiates the gene transcription process, which is essential for cell growth and division. (**B**) In the case when abundant BRCA1 or supplemented BRCA1 mimetics are in the breast cancer cells, BRCA1 will act as a Corepressor and replace Cyclin D1 from the 17-β estradiol liganded ERα complex at ERE. This complex downregulates the gene transcription, which is essential for cell growth and cell division.

Every year, there are about 2.3 million instances of breast cancer globally, making it the most prevalent disease among people [1,2,3]. In 95% of nations, breast cancer ranks as the primary or secondary cause of mortality among females [4]. The World Health Organization (WHO) has unveiled a new Global Breast Cancer Initiative Framework (GBCIF) aimed at saving 2.5 million lives from breast cancer by 2040. It advises countries to adopt the three pillars of health promotion, early detection, timely diagnosis, and comprehensive management, to achieve these objectives. Estimates suggest that one in eight women, or around 12%, may end up diagnosed with breast cancer throughout their lifetime. The BRCA1 gene encodes the BRCA1 protein, which functions as a tumor suppressor. BRCA1 inhibits excessive or uncontrolled cellular proliferation and division [5,6,7,8,9]. A mutant BRCA gene may become ineffective in repairing damaged DNA and preventing breast cancer [5,10,11,12,13,14,15]. Estimates show that before age 70, 55–65% of women with the BRCA1 mutation will develop breast cancer. Consequently, individuals with a BRCA1 gene mutation will have an increased likelihood of developing breast cancer and are prone to manifesting the disease at a younger age. The carrier of the mutated gene may transmit the gene mutation to their progeny. Studies have demonstrated that BRCA1 inhibits ERα activity, in part, through direct interactions between its amino-terminal domains and the carboxyl-terminal region of ERα. Additionally, immunoprecipitation experiments [16] indicate that cyclin D1 requires ERα residues 282 to 378 for binding in cultured cells. The same segment of ERα, residues 338–379, required for interaction with cyclin D1, was likewise sufficient for interaction with BRCA1. The same area of ERα for cyclin D1 or BRCA1 binding suggests that cyclin D1 and BRCA1 may compete for interaction with Erα [17,18] (Figure 1). The distinctive interactions of BRCA1 with ERα [19] further corroborate the idea of our study (Table 1). Utilizing high-resolution mapping, we proposed specific interaction sites within a three-dimensional model of the BRCA1:ER-α (partial) complex. Based on this model, we identified a series of coumarin nucleus-based compounds capable of occupying a predicted BRCA1-binding cavity within ERα, thereby mimicking BRCA1’s ability to suppress ERα activity. Notably, these BRCA1 mimetics are tiny compounds that do not interact with the ligand-binding pocket of ERα and operate through a distinct mechanism from selective estrogen receptor modulators (SERMs) and degraders (SERDs). BRCA1 mimetics have the potential to overcome resistance to hormonal therapies by directly modulating the estrogen receptor, reducing estrogen signaling, and enhancing tumor suppression pathways. This may help lower the risk of resistance-associated mutations. In contrast, resistance to SERMs and SERDs often arises due to ESR1 mutations or the activation of alternative growth pathways. Additionally, BRCA1 mimetics could be combined with PARP inhibitors, chemotherapy, or hormonal therapies to improve treatment efficacy, particularly in tumors with BRCA1 dysfunction. While SERMs and SERDs remain the cornerstone of ERα-positive breast cancer treatment, BRCA1 mimetics offer a novel approach by targeting tumor suppressor pathways beyond estrogen signaling. This strategy could be especially beneficial for patients with BRCA1 mutations or those resistant to endocrine therapy, providing a complementary or alternative therapeutic option. This study presents the synthesis, characterization, and biological evaluation of these novel compounds. The results indicate that BRCA1 may impede the connection of cyclin D1 with ERα, suggesting a possible mechanism for the suppression of ERα [20,21,22]. Coumarin nuclei have been reported to exhibit a strong affinity for Erα [21]. Based on this, we designed molecular hybrids with a coumarin nucleus coupled to a substituted thiosemicarbazone chain. This structural framework was designed to increase conformational flexibility, maximizing molecular adaptability and binding efficiency inside the active site [23]. This research seeks to identify a new class of BRCA1 mimetics for ER-positive breast cancer that operates differently from traditional antiestrogens. Twenty-four coumarin thiosemicarbazone hybrids were synthesized after their novelty verification by SciFinder. Cytotoxicity assays were performed on MDA MB-231 cell lines (BRCA1 mutant), then followed by gene expression analysis for the genes cyclin D1 and BCl2 to corroborate our hypothesis. The rationale for choosing Cyclin D1 and BCL2 genes for gene expression research is that overexpression of cyclin D1 and BCL2 has been reported in over 50% of human breast cancers of all histological types [24,25,26]. Cyclin D1 and BCL2 are the two key genes regulated by ERα. Cyclin D1 and BCL2 gene overexpression is identified in the earliest stages of breast cancer development, such as ductal carcinoma in situ, and is maintained in all phases of metastasis [27]. The upregulation of cyclin D1 and BCL2 via ERα signaling correlates with an enhanced proliferative response in breast cancer cells [28,29]. This work used glyceraldehyde-3-phosphate dehydrogenase (GAPDH) as a reference gene, serving as a housekeeping gene for precise sample normalization to detect minor but significant variations in expression across the samples [30,31,32,33,34,35,36,37]. The delta-delta Ct (∆∆Ct) approach, referred to as the 2^−∆∆Ct^ method (where ‘Ct’ denotes the cyclic threshold of the sample), was used in qRT-PCR to ascertain the relative fold gene expression of the samples [38]. Molecular docking experiments were performed to examine the protein–ligand interactions with the BRCA1 binding domain (AA 338–387) of ERα (PDB ID-1A52), using NSC35446 (BRCA1 mimetic) as a reference [22].

## 2. Experimental Section

### 2.1. Materials

All the starting materials, chemicals, reagents, and solvents were of reagent grade, purchased from Sigma Aldrich (Bangalore, India), and used without further purification. The purity of the products was assessed, and the reactions were monitored using TLC analysis. For thin-layer analytical chromatography, we used 60F_254_ (0.5 mM) MERCK aluminum-backed pre-coated silica gel plates. The IR spectra were obtained using the Perkin-Elmer FT-IR spectrometer (Thane, India). BRUKER (400MHz FT-NMR) (Billerica, MA, USA) in DMSO solvent was used to obtain the ^1^H -NMR and ^13^C -NMR spectra, with TMS serving as an internal standard. The compounds’ mass spectra were ascertained using Shimadzu LC-MS (Kyoto, Japan).

#### Synthesis of *N*-(Substitute Benzylidene)-2-((*E*)-7-hydroxy-4-methyl-2*H*-chromen-2-ylidene) Hydrazine-1-carbothioamide (**7**) (Scheme-I)/*N*-(Substitute Benzylidene)-2-((*E*)-4-hydroxy-2*H*-chromen-2-ylidene)hydrazine-1-carbothioamide (**9**) (Scheme-II)

**Figure 2:**
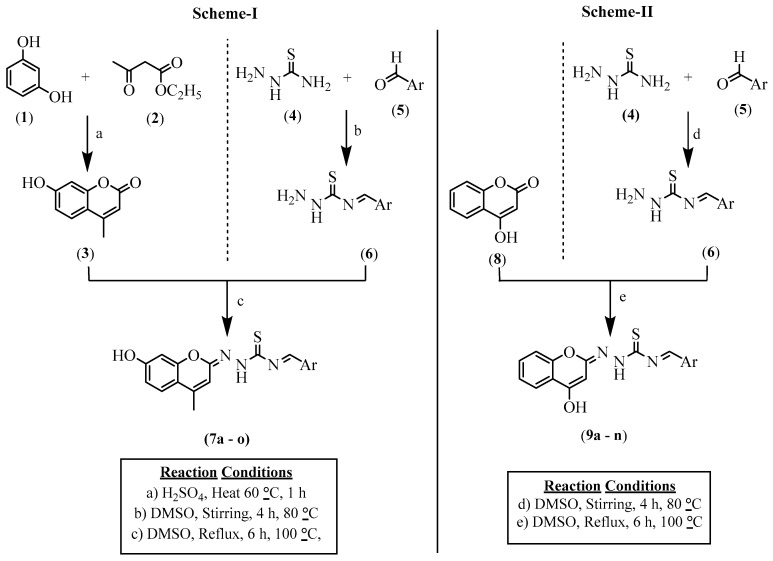
Route followed in order to obtain coumarin thiosemicarbazide derivatives **7a-o** and **9a-n**.

A total of twenty-four coumarin thiosemicarbazone derivatives were prepared by following the reported method [21,22]. Substituted benzaldehydes (0.01 mol) dissolved in DMSO (50 mL) were mixed with thiosemicarbazide (0.01 mol, 0.91 g) and stirred for 4 h at 80 °C. After completing, the reaction mixture was moved to ice-cold water, where (E)-N-benzylidenehydrazine carbothioamide (**6**) solidified as a residue. It was then vacuum-filtered and allowed to dry. This intermediate (**6**) was common to all compounds being synthesized from Schemes I and II (Figure 2). The intermediate (**6**) (0.01 mol) in DMSO (50 mL) was mixed with 7-hydroxy 4-methyl coumarin (0.01 mol) for Scheme-I and 4-hydroxy coumarin (0.01 mol) for Scheme-II and stirred for 4 h at 60 °C. After completing, the reaction mixture was moved to ice-cold water. The *N*-(substitute benzylidene)-2-((*E*)-7-hydroxy-4-methyl-2*H*-chromen-2-ylidene) hydrazine-1-carbothioamide (**7**) (Scheme-I)/*N*-(substitute benzylidene)-2-((*E*)-4-hydroxy-2*H*-chromen-2-ylidene)hydrazine-1-carbothioamide (**9**) (Scheme-II) solid precipitate was formed, vacuum-filtered, dried, and its melting point was ascertained.

### 2.2. Pharmacology

#### 2.2.1. Cytotoxicity Screening

Vero cells are known for their ability to assess the safety of new compounds and their sensitivity to a wide range of toxic substances. MDA-MB-231 cells are derived from human ER-positive breast cancer tissue. MDA-MB-231 cells provide a reliable method for assessing the toxicity and effectiveness of potential anticancer compounds, especially those that target estrogen receptors or associated pathways. We assessed the anticancer activity of the compounds on Vero cells and MDA-MB-231 using the MTT (3-(4,5-dimethyl thiazol-2yl)-2,5-diphenyl tetrazolium bromide) assay [21]. The MTT assay is one of the most commonly used for cancer drug screening [50,51]. This is a basic and easy assay for cytotoxicity, and it is one of the most popular cytotoxicity assays in the research laboratory [52]. We plated cells (1 × 10^5^/well) in 96-well plates using 0.2 mL of medium per well. We carefully removed the media from the wells for the MTT assay after incubation. After two to three MEM (*w*/*o*) FCS washes, 200 µL of MTT (5 mg/mL) was added to each well. We incubated the plates for 6–7 h in a 5% CO^2^ incubator to test for cytotoxicity. Following the incubation, we added 1 mL of vehicle (DMSO) to each well, mixed it thoroughly with a micropipette, and left it for 45 s. NADPH-dependent dehydrogenase catalyzes the conversion of tetrazolium salt (MTT) into a purple formazan product in cells. This indicates the presence of metabolically active cells. We placed the solution in the spectrophotometer’s cuvette and recorded the optical density at 595 nm, using DMSO as the blank reference. Measurements were conducted, and the concentration required for 50% inhibition of viability (IC_50_) was visually determined. A standard graph was constructed, with the drug concentration plotted on the *X*-axis and relative cell viability on the *Y*-axis.

$$\%Cell\;Viability=\frac{Mean\;Optical\;Density\;of\;Individual\;Sample}{Mean\;Optical\;Density\;of\;Control}\times 100$$

#### 2.2.2. The Construction of Protein–Protein Interaction Network (PPI) Associated with ESR1

We performed a protein–protein interaction (PPI) analysis using the STRING database [53,54]. STRING is a database that compiles known and predicted protein–protein interactions, encompassing both direct (physical) interactions and indirect (functional) associations. All the interactions between them were derived from high-throughput lab experiments, previous knowledge in curated databases at a high level of confidence, and from lab experiments, curated databases, and gene expression data, with the same confidence to construct the PPI network with the co-expression interactions for comparison.

#### 2.2.3. Analysis of Gene Expression for Cyclin D1 and BCL2 with RT-qPCR

To determine whether the synthesized compounds were repressing the ER alpha protein or not, we performed gene expression analyses for Cyclin D1 and BCL2 using the real-time quantitative polymerase chain reaction (RT-qPCR). RT-qPCR is an efficient, simple, and low-cost technique to quantify gene expression levels. The delta-delta Ct (ΔΔCt) method is a widely used technique for analyzing real-time quantitative PCR (qPCR) data. We chose three compounds, **9b**, **9l**, and **9m**, based on their lowest IC_50_ values and conducted gene expression studies for BCL2 and Cyclin D1. We used the GAPDH as a reference gene. We chose primer pairings with the lowest penalty value designed by the Primer Express 2.0 software to obtain the best results [38].

##### Real-Time PCR by ΔΔCt Method

A 100-picomolar primer stock was prepared utilizing primers of Cyclin D1, BCL2, and GAPDH (Table 6), which were then diluted to a 10-picomolar working stock for use in real-time PCR. RNA was extracted from four samples utilizing the PureLink RNA Mini Kit and quantified using the Biophotometer Plus (Table 3). A total of 1 µg of RNA was taken from the isolated RNA and used for cDNA synthesis with the Takara Prime Script 1st Strand cDNA Synthesis Kit. Each real-time PCR experiment utilized 25 ng of cDNA, 10 picomolar forward and reverse primers, and a final reaction volume of 20 µL. Cyclin D1, BCL2, and GAPDH average Ct values were extracted from each sample’s replicates. Then, using the formula below (1), we determined the ΔCt from the mean Ct values of Cyclin D1 and BCL2 with reference to the reference gene’s GAPDH mean Ct values. In the real-time qPCR, the temperature was set at 95 °C for 10 min (denaturation), then 40 cycles at 95 °C for 15 s and 60 °C for one minute. Each sample was processed in triplicate in six-well plates for the qPCR analysis using the Qiagen kit. The comparative Ct was used for quantification analysis, and the Ct values (Ct1, Ct2, and Ct3) were averaged.

ΔCt = Ct (gene of interest) − Ct (housekeeping gene)(1)

We calculated the ΔΔCt from the ΔCt values of the Cyclin D1 and BCL2. The ΔCt values of GAPDH were calculated using the following Formula (2).

ΔΔCt = ΔCt (Sample) − ΔCt (Control)(2)

Finally, we calculated the fold gene expression for CyclinD1 and BCL2 with reference to the reference gene GAPDH using the following Formula (3) (Table 5).

Fold gene expression = 2^−ΔΔCt^ (3)

### 2.3. In Silico Studies: Molecular Docking, MMGBSA, and ADME Studies

Three ligands were selected for the molecular docking and MMGBSA studies to analyze the protein–ligand interactions and binding energies, respectively, at the BRCA1 binding pocket (AA 338–387) of ERα, based on the in vitro screening results and gene expression studies. Ligprep 2020-2 was utilized to energetically minimize the ligands for the input structure. The optimized ER alpha protein (PDB ID: 1A52) was prepared utilizing the protein preparation wizard (Epik v4.1, Schrodinger suite 2020-2). The molecular docking and MMGBSA were performed utilizing Glide and Prime Integrated Maestro 11.3 in accordance with the methodology outlined in the literature [55,56]. Prime MMGBSA computes the energy of the optimized free receptor, the free ligand, and the ligand–receptor complex. It also computes the ligand strain energy by positioning ligands in a solution set up by the VSGB 2.0 suite. ADME characteristics of the ligands were predicted in silico using the QikProp (v5.3) module of Schrodinger, using the Lipinski rule of five (RO5). We evaluated the ionization potential (IP in eV), human oral absorption (HOA), topological polar surface area (TPSA) [57], skin permeability (QPlogKp), and the logarithm of the compound partition coefficient between water and gas (QPlogPw), as well as between octanol and water (QPlogPo/w) [58,59]. Solubility (QPlogS) [60] is an important factor in the development of novel compounds and was calculated. An important parameter in forecasting bioavailability and the passive transport of an active substance was predicted via the blood/brain partition coefficient (QPlogBB) [61].

## 3. Results and Discussion

### 3.1. Chemistry

#### *N*-((*E*)-Benzylidene)-2-((*E*)-7-hydroxy-4-methyl (Scheme-I)/4-Hydroxy-2*H*-chromen-2-ylidene) Hydrazine-1-carbothioamides (Scheme-II) Derivatives

A total of twenty-four compounds were synthesized using two diverse schemes. Thiosemicarbazide reacts with aromatic aldehydes/substituted benzaldehydes in a nucleophilic addition mechanism. The nucleophilic addition reaction with 7-hydroxy 4-methyl coumarin (Scheme-I)/4-hydroxy coumarin (Scheme-II) yields aromatic substituted thiosemicarbazones, which, in turn, produced *N*-((*E*)-benzylidene)-2-((*E*)-7-hydroxy-4-methyl-2*H*-chromen-2-ylidene) hydrazine-1-carbothioamide. The derivatives were recrystallized with ethanol and produced yields ranging from 65 to 90%. The structures of the synthesized compounds were confirmed with spectral data (^1^H-NMR, ^13^C-NMR, FT-IR, and MASS) and are presented in the Supplementary Data.

### 3.2. Pharmacology

#### 3.2.1. In Vitro Cytotoxicity Assay

The developed compounds were screened for cytotoxicity on Vero and MDA-MB-231 cells using the MTT test; the findings are displayed in Table 2. Using MDA-MB-231 cells, the title compounds demonstrated mild to moderate in vitro cytotoxicity. Compound **9b** shows almost similar IC_50_ values (14.49 µM) as the reference drug raloxifene IC_50_ value (13.7 µM). Vero cells exhibited minimal cytotoxicity at higher concentrations of the synthesized compounds compared to the MDA-MB-231 cells, indicating a favorable cytotoxicity profile. For the gene expression experiments, four of the synthesized compounds with the lowest IC_50_ values, **9b** (14.49 µM), **9l** (35.0 8 µM), and **9m** (42.12 µM), were selected (Table S1. Supplementary Data).

#### 3.2.2. The Construction of Protein–Protein Interaction Network (PPI) Associated with ESR1

**Figure 3:**
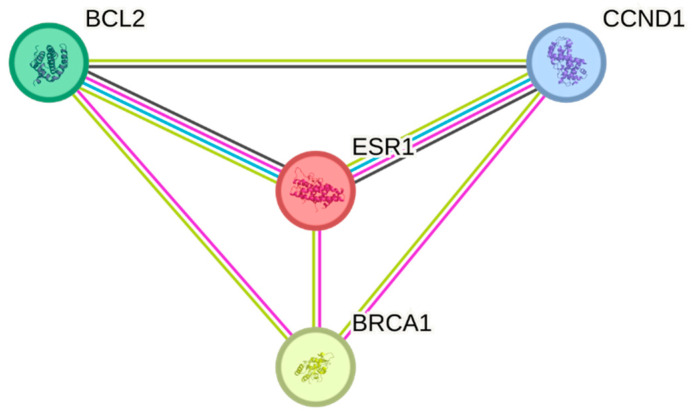
String protein–protein interaction network. (The network nodes (colored circles) represent proteins, with a single node representing all the proteins produced by a single protein-coding gene. Colored lines between the nodes (edges) indicate the different types of interaction evidenced by fusion genes (pink lines), neighborhood of genes (green lines), co-occurrence across species (blue line), experimental evidence (purple line), and co-expression in the same or other species (black line)).

To find the relation between the ESR1, BRCA1, Cyclin D1, and BCL2 genes, we constructed a STRING PPI network. In the string output, individual proteins are seen as nodes, and their interactions are seen as edges that are color-coded according to their nature. Figure 3 shows the PPI analysis of the three genes that were composed of three nodes (one for each protein) and 14 edges linking them, a number of edges higher than that expected for random associations, which indicated that these proteins were biologically connected.

#### 3.2.3. Gene Expression Studies

**Figure 4:**
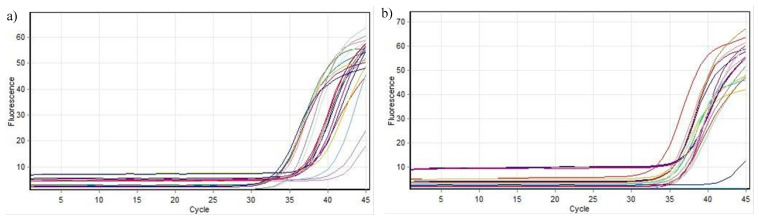
Fluorescence acquisition data: (**a**) RT-qPCR fluorescence amplification curves of CyclinD1 transcript and (**b**) RT-qPCR fluorescence amplification curves of BCL2 transcript.

Based on the in vitro findings, compounds **9b**, **9l**, and **9m** were selected for the gene expression analyses of Cyclin D1 and BCL2 using real-time PCR with the ∆∆Ct method. Fluorescence acquisition data depicted RT-qPCR amplification curves for Cyclin D1 and BCL2, with GAPDH serving as an internal control for normalization and relative quantification (Figure 4). The fluorescence signal generated during qPCR is directly proportional to the amount of synthesized DNA (Table 3, Table 4, Table 5 and Table 6), enabling visualization through amplification plots.

**Table 3:** List of genes amplified, relative primers, and main pathways.

S. No.	Gene	Forward Primer (5′-3′) Reverse Primer (3′-5′)	Primer Conc. (µM)	Annealing Temp. (°C)	Amplicon Size (bp)
1	Cyclin D1	**5′-**CGGGATCCCCAGCCATGGAACACCAGC**-3′** **3′**-CGGAATTCGCGCCCTCAGATGTCCACG-**5′**	0.05	58	75
2	BCL2	**5′-**CTGGTCCAAGAGGATTTCCA**-3′** **3′**-TCATTGCCTTGCACGTAGAG-**5′**	0.05	58	100
3	GAPDH	**5′-**ATGGCATTCCGTGTTCCTAC**-3′** **3′**-CCTTCAACTTGCCCTCTGAC-**5′**	0.05	58	117

**Table 4:** The concentration of RNA extracted from MDA-MB-231 cells and the concentration of cDNA.

S. No	Sample	A260/280	RNA Concentration (µg/mL)	A260/280	cDNA Concentration (µg/mL)
1	**9l**	2.04	311.8	1.82	637.4
2	**9b**	2.11	297.2	1.77	595.6
3	**9m**	1.89	428.3	1.71	629.3
4	MDA-MB-231 Cell Control	2.16	521.4	1.82	552.8

**Table 5:** Ct mean and ΔΔCt values of Cyclin D1 gene in MDA-MB-231 cells. (Supplementary Data: Tables S2 and S3).

S. No		Cyclin D1	GAPDH			
		Ct Mean	Ct Mean	Δ Ct	ΔΔCt	2^(−ΔΔCt)^
1	MDA-MB-231 Cell Control	27.82	33.38	−5.56	0	0
2	**9l**	31.62	34.98	−3.36	2.2	−0.217637640
3	**9b**	28.85	34.98	−6.13	−0.57	1.484523570
4	**9m**	31.45	34.98	−3.53	2.03	−0.214855074

**Table 6:** Ct mean and ΔΔCt values of BCL2 gene in MDA-MB-231 cells.

S. No		BCL 2	GAPDH			
		Ct Mean	Ct Mean	Δ Ct	ΔΔCt	2^(−ΔΔCt)^
1	MDA-MB-231 Cell Control	30.13	31.2	−1.08	0	0
2	**9l**	33.42	30.7	2.72	1.64	−0.320856473907
3	**9b**	31.04	30.7	0.34	1.42	−0.3737123121587
4	**9m**	31.03	30.7	0.33	1.41	−0.3763116868527

**Figure 5:**
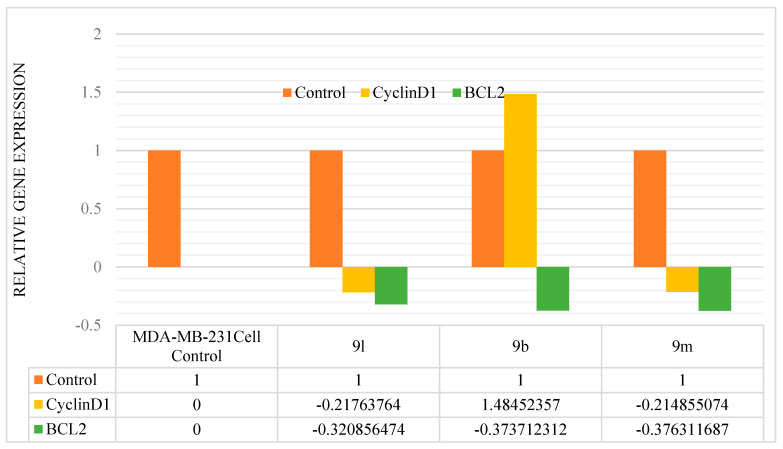
Relative gene expression of Cyclin D1 and BCL2 in MDA-MB-231.

The gene expression data indicate that, except for **9b**, compounds **9l** (−0.217) and **9m** (−0.214) downregulate CyclinD1. All three compounds downregulated the BCL2 gene, with specific repression observed for **9b** (−0.373), **9l** (−0.320), and **9m** (−0.376) (Table 7 and Figure 5).

### 3.3. In Silico Studies: Molecular Docking, MMGBSA, and ADMET Studies

**Figure 6:**
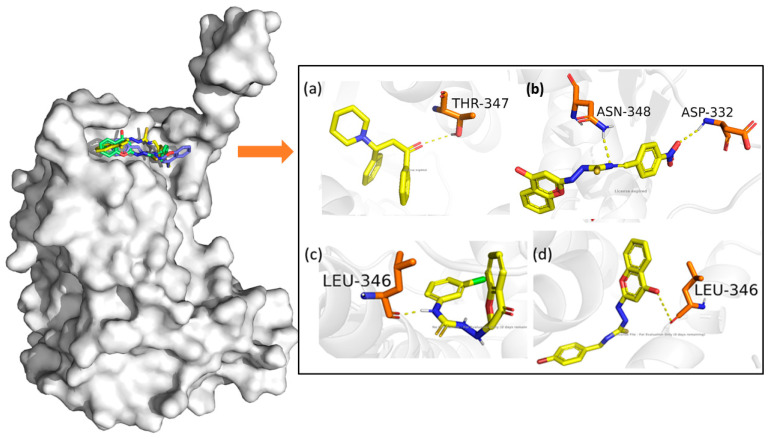
Three-dimensional protein–ligand H bond interactions. (**a**) NSC35446 (BRCA1 Mimetic): Thr347; (**b**) **9l**: Asn348 and Asp332; (**c**) **9b**: Leu346; and (**d**) **9m**: Leu346. **NSC35446** and our synthesized ligands at the BRCA1 binding region (AA 338–387) of ERα.

The glide g scores were for **9b** (−10.268), **9l** (−9.854), and **9m** (−10.569) given in Table 8. Figure 6 illustrates the most significant interactions involving hydrogen bonds [63]. Compound **9b** interacted with Asn348 and Asp332; **9l** interacted with Asp346; and **9m** interacted with Leu346.

Table 10 presents the results of the pharmacokinetics studies (ADME) conducted through computational analysis of the compounds. The synthesized compounds exhibit suitable ADME values that fall within the established range, with no violations of the Lipinski rule of five. The compounds **9b**, **9l,** and **9m** exhibit a favorable QPlogPw (octanol/water) value for biological effectiveness. Furthermore, the pharmacological properties observed are significant, with 95% of the examined medicines demonstrating effective absorption by the body and an absence of harmful effects. Every ligand possesses a molecular weight that aligns with the acceptable range, and the LogS data are accessible for 95% of the medicines currently on the market.

For acute oral toxicity, compounds **9b**, **9l**, and **9m** are categorized as toxicity class IV; their estimated LD_50_ values are 560 mg/kg, 500 mg/kg, and 826 mg/kg, respectively (Table 11). Immunotoxicity was expected to be active with confidence values of 0.90, 0.98, and 0.96 for **9b**, **9l**, and **9m,** respectively, while hepatotoxicity was anticipated to be with confidence levels of 0.76 (active), 0.60, and 0.56. Furthermore, the mutagenicity projections for **9b**, **9l**, and **9m** produced confidence values of 0.51, 0.64, and 0.50, respectively. Toxicity predictions using the ProTox-II platform indicate that, apart from immunotoxicity, all three compounds exhibit a relatively lower toxicity profile with respect to acute oral toxicity, hepatotoxicity, and mutagenicity.

The LD_50_ is the median lethal dose, meaning that 50% of test subjects die upon exposure to a compound. The globally harmonized system (GHS) classified various chemicals based on toxicity types, such as Class I: fatal if swallowed (LD_50_ ≤ 5), Class II: fatal if swallowed (5 ≤ LD_50_ ≤ 50), Class III: toxic if swallowed (50 ≤ LD_50_ ≤ 300), Class IV: harmful if swallowed (300 ≤ LD_50_ ≤ 2000), Class V: may be harmful if swallowed (2000 ≤ LD_50_ ≤ 5000), and Class VI: non-toxic (LD_50_ ≥ 5000).

**Figure 7:**
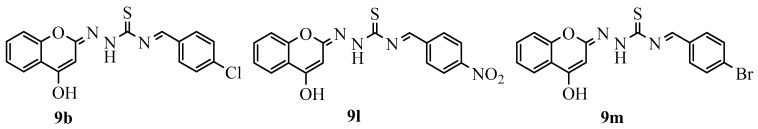
Chemical structures of **9b**, **9l**, and **9m**.

The findings demonstrate several structural features suggestive of the BRCA1 mimetic property: the presence of the coumarin ring, a hydroxyl group substitution at the fourth position of the coumarin nucleus, the absence of the keto group, the introduction of an imine group at the second position of the coumarin nucleus, and the presence of thiosemicarbazone with a para (electronegative) substituted aromatic ring, Figure 7.

In this research, we primarily concentrated on in silico analyses, cytotoxicity assays, and gene expression evaluations to assess the potential anticancer properties of our compounds. However, we recognize the importance of further validation to strengthen our findings. In our future investigations, we intend to delve deeper into the underlying mechanisms of action. This will involve conducting detailed apoptosis assays to investigate the programmed cell death pathways, as well as a comprehensive cell cycle analysis using flow cytometry to determine how our compounds influence cellular proliferation and cycle progression. Additionally, we plan to utilize Western blotting techniques to quantify the protein expression associated with key signaling pathways involved in cancer progression and responses to treatment. Furthermore, we aim to incorporate wound healing assays to evaluate the effects of our compounds on cell migration and their ability to impede metastatic behavior.

## 4. Conclusions

A total of 24 (**7a–l** & **9a–n**) coumarin thiosemicarbazone hybrids were synthesized from 7-hydroxy 4-methyl coumarin/4-hydroxy coumarin and thiosemicarabazide with different aldehydes using two schemes and were characterized. **9b**, **9l**, and **9m** have optimum binding interactions with residues at the BRCA1 binding pocket of ERα and have significant docking scores. The MMGBSA analysis revealed stronger binding of the ligands to the protein. The molecular ADME and in silico toxicity prediction profile estimation indicated the safety of these compounds. In vitro, the chemicals’ anticancer efficacy against the MDA MB 231 cell lines ranged from modest to high. According to the in silico, in vitro, and gene expression investigations, the compounds **9b**, **9j**, and **9l** are useful inhibitors of ERα-positive breast cancer. **9b**, **9j**, and **9l** (BRCA1 mimetics) can function as ERα corepressors in breast cancer cells, mimicking the activity of the BRCA1 protein. These compounds can displace Cyclin D1 from the 17β-estradiol-liganded ERα complex at the estrogen response element (ERE), resulting in the downregulation of key target genes, including **Cyclin D1** and **BCL2,** which are critical for cell proliferation and survival. This mechanism ultimately suppresses tumor growth by inhibiting the transcriptional activation of the genes essential for cell cycle progression and division. These compounds can then be investigated further and utilized as a parent core molecule to create some novel lead molecules.

## Figures and Tables

**Table 1:** BRCA1 unique interactions with ERα [39].

S. No	Experimental Evidence Code	Direction of Interaction	Dataset
1	Affinity Capture-Western	BAIT/HIT	Fan S (2001) [17]
		BAIT/HIT	Kawai H (2002) [40]
		BAIT/HIT	Fan S (2002) [41]
		HIT	Nakuci E (2006) [42]
		BAIT	Wang C (2005) [43]
		BAIT/HIT	Dizin E (2010) [44]
		BAIT/HIT	Ma Y (2010) [45]
		BAIT/HIT	Ma YX (2005) [18]
		BAIT/HIT	Ma Y (2007) [46]
		HIT	Jung YS (2014) [47]
2	Biochemical Activity	BAIT	Stewart MD (2017) [48]
3	Co-localization	BAIT	Zheng L (2001) [49]
4	Reconstituted Complex	BAIT	Fan S (2001) [17]
		HIT	Wang C (2005) [16]
		HIT	Ma YX (2005) [18]
		BAIT/HIT	Kawai H (2002) [40]

**Table 2:** Results of the cytotoxicity assay of twenty-four synthesized coumarin thiosemicarbazone analogs. (Raloxifene was taken as a reference drug [62] to compare the IC_50_ values of the synthesized compounds).

S. No	Compound	VERO (µM)	MDA MB 231 (µM)
1	**7a**	513.18	357.31
2	**7b**	165.31	70.46
3	**7c**	1631.29	307.69
4	**7e**	775.56	409.09
5	**7h**	148.69	80.30
6	**7i**	291.55	237.05
7	**7j**	213.64	151.33
8	**7k**	234.80	151.93
9	**7l**	293.44	353.27
10	**7m**	149.32	90.49
11	**7n**	405.19	189.61
12	**7o**	257.53	136.98
13	**9a**	293.36	250.00
14	**9b**	60.86	14.49
15	**9c**	87.37	61.48
16	**9e**	135.29	79.41
17	**9f**	200.96	239.70
18	**9g**	283.42	342.24
19	**9i**	450.49	222.77
20	**9j**	429.79	232.09
21	**9k**	252.63	278.94
22	**9l**	87.719	35.08
23	**9m**	68.73	42.12
24	**9n**	119.30	47.72
Standard	Raloxifene [62]	-	13.7

**Table 7:** Relative gene expression of CyclinD1 and BCL2.

S. No	Compound	Control	CyclinD1	BCL2
1	MDA-MB-231Cell Control	1	-	-
2	**9l**	1	−0.217637640	−0.3208564740
3	**9b**	1	1.484523570	−0.3737123121
4	**9m**	1	−0.214855074	−0.3763116868

**Table 8:** Molecular docking parameters using Glide.

S. No	Compound	Glide g Score	XP G Score	Glide vdW	Glide Columb	Glide Energy
1	**9b**	−10.268	−10.268	−45.316	−2.369	−47.685
2	**9l**	−9.854	−9.854	−38.549	−3.247	−41.796
3	**9m**	−10.569	−10.569	−46.786	−2.648	−49.434

Prime MMGBSA analysis revealed the binding energy ΔG of **9b** with −52.99 Kcal/mol, **9l** with −42.58 Kcal/mol, and **9m** with −48.47 Kcal/mol (Table 9).

**Table 9:** MMGBSA binding energies using Prime.

**S. No**	**Compound**	**MMGBSA dG Bind**	**MMGBSA dG Bind Coloumb**	**MMGBSA dG Bind Covalent**	**MMGBSA dG Bind H Bond**	**MMGBSA dG Bind vdW**
1	**9b**	−52.99	10.36	7.56	0.45	−71.36
2	**9l**	−42.58	9.54	9.21	1.26	−62.59
3	**9m**	−48.47	10.72	6.32	1.05	−66.56

**Table 10:** ADMET predictions using QuickProp.

Compound	QPlogPw	QPlogPo/w	QPlogS	QPlogBB	QPlogKp	IP(eV)	HOA	TPSA	RoF
**9b**	12.74	3.574	−5.454	−0.297	−1.415	8.524	3	80.213	0
**9l**	13.687	2.745	−4.466	−0.411	−1.909	8.244	3	79.027	0
**9m**	11.95	3.744	−4.696	−0.281	−1.973	8.426	3	71.469	0
Std Range	4 to 45	2 to 6.5	−6.5 to 0.5	−3 to 1.2	−8 to −1	−7.9 to 10.5	−1.5 to 1.5	7 to 200	0 to 4

QPlogPw—water/gas partition coefficient; QPlogPo/w—octanol/water partition coefficient; QPlogS—aqueous solubility; QPlogBB—blood/brain partition coefficient; QPlogKp—skin permeability; IP(eV)—ionization potential; HOA—human oral absorption; TPSA—topological polar surface area; RoF—Lipinski’s rule of five.

**Table 11:** Toxicity prediction using the ProTox-II platform.

Compound	Predicted LD_50_ (mg/Kg)	Hepatotoxicity	Immuno Toxicity	Mutagenicity
**9b**	560 (Class IV)	0.76 (Active)	0.90 (Active)	0.51
**9l**	500 (Class IV)	0.60	0.98 (Active)	0.64
**9m**	826 (Class IV)	0.56	0.96 (Active)	0.50

## Data Availability

The original contributions presented in this study are included in the article/Supplementary Material. Further inquiries can be directed to the corresponding author.

## Supplementary Materials

The following supporting information can be downloaded at: https://www.mdpi.com/article/10.3390/life15040581/s1, Images of analytical spectra with characterization, In vitro cytotoxicity microscopic images, Real-Time PCR Raw Data for Cycling A. Green Ct Values for Cyclin D1, Real-Time PCR Raw Data for Cycling A. Green Ct Values for Bcl2 gene expression raw data, and in silico files are attached.

## Abbreviations

qRTPCR—Quantitative Real-Time Polymerase Chain Reaction, MLR—Multiple Linear Regression, BRCA1—Breast Cancer Susceptibility Protein 1, ERα—Estrogen Receptor Alpha, PDB—Protein Data Bank, MMGBSA—Molecular Mechanics/Generalized Born Surface Are
