# Supplementary material for: A New Class of BRCA1 Mimetics for ERα-Positive Breast Cancer Therapy: Design, Synthesis, In Silico Screening, In Vitro Assay, and Gene Expression Analysis
Source: Life (Basel). 2025 Apr 1;15(4):581. doi: 10.3390/life15040581 (PMC12028564; doi:10.3390/life15040581)
Supplement: Supplementary file 1 [file life-15-00581-s001.zip › life-3514109-supplementary.pdf]

Supporting Information

A New Class of BRCA1 Mimetics for ERα-Positive Breast Cancer Therapy: Design, Synthesis, In Silico Screening, In Vitro Assay, and Gene Expression Analysis

CHEMISTRY

Scheme I & Scheme II

The thiosemicarbazone coumarin derivatives were synthesized in Scheme I. Thiosemicarbazide participates in nucleophilic addition reactions with various substituted benzaldehydes. Substituted thiosemicarbazones are generated, which subsequently participate in a nucleophilic addition reaction with 7-Hydroxy 4-methyl coumarin, resulting in the formation of *N*-((*E*)-benzylidene)-2-((*E*)-7-hydroxy-4-methyl-2*H*-chromen-2-ylidene) hydrazine-1-carbothioamide after 6 hours of reflux in DMSO solvent. The derivatives produced yields ranging from 65% to 90% and underwent recrystallization using ethanol. The confirmation of the structure of (*E*)-*N*-benzylidenehydrazine carbothioamide was achieved through the unique peaks observed in the IR and Mass spectra. The formation of **7a** was confirmed by the disappearance of the C=O bond at 1721 cm^{-1} and the emergence of the characteristic C=S stretch and C=N stretch at 1049 cm^{-1} and 3121.18 cm^{-1}, respectively, in the infrared (IR) spectra. The ^{1}H NMR analysis of **7a** indicated the presence of an NH proton singlet at 10.80 ppm, suggesting that the C=O group was substituted by an NH_2 group from the thiosemicarbazide. The signals indicated the synthesis of **7a** at 154.93 and 159.96 in ^{13}C NMR, attributed to CNH bonds, and at 174.90 due to the C=S bond. The presence of a M+1 peak at 415.2001 in the mass spectra provides additional support for this finding.

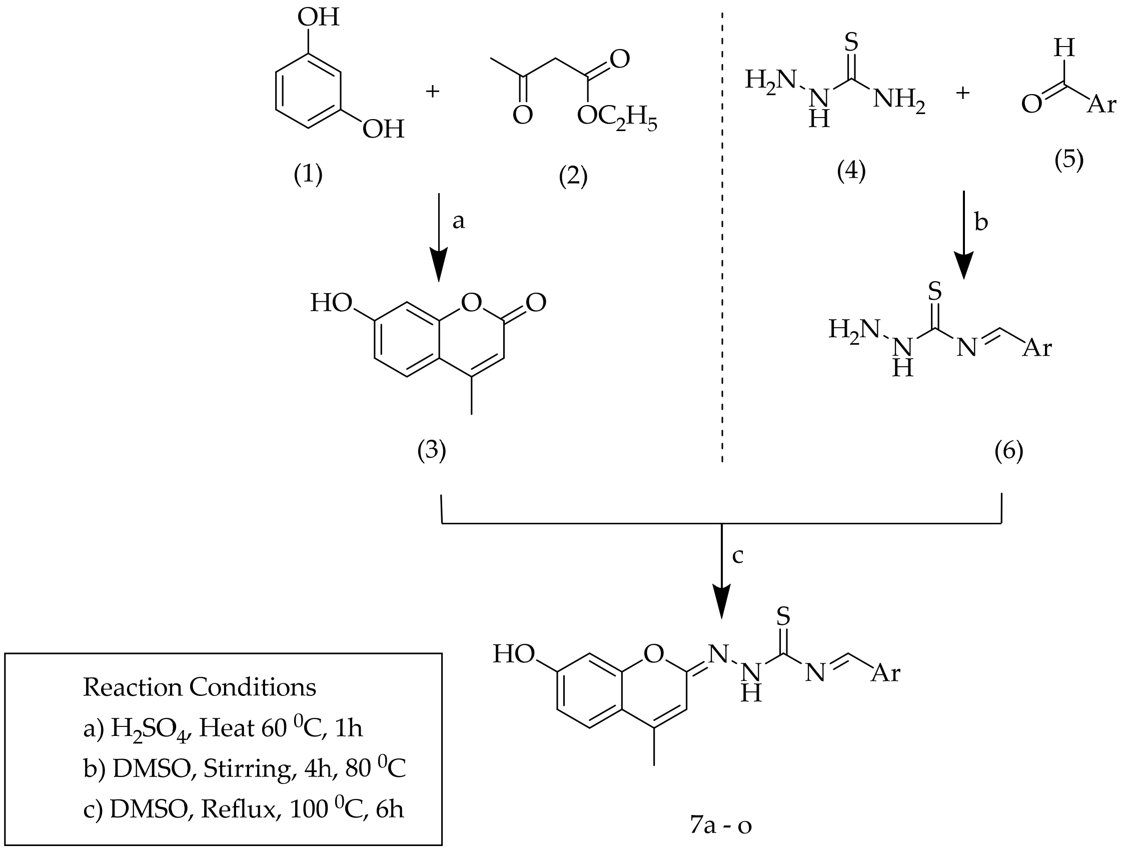

Reaction Conditions
a) H_2SO_4, Heat 60 ^{0}C, 1h
b) DMSO, Stirring, 4h, 80 ^{0}C
c) DMSO, Reflux, 100 ^{0}C, 6h

Figure S1. Scheme I. Synthesis of *N*-((*E*)-benzylidene)-2-((*E*)-7-hydroxy-4-methyl-2*H*-chromen-2-ylidene) hydrazine-1-carbothioamide

Spectral Characterizations

***N*-((*E*)-2,4-dichlorobenzylidene)-2-((*E*)-7-hydroxy-4-methyl-2*H*-chromen-2-ylidene) hydrazine-1-carbothioamide (7a)**

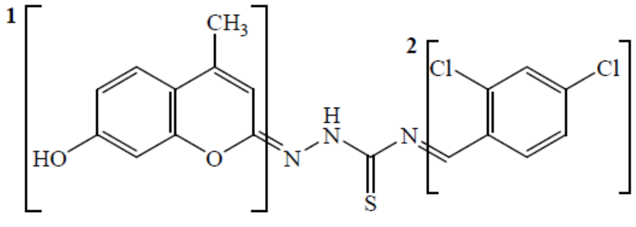

Solid gray color; Rf value of 0.78; yield achieved is 71%. M.P: 91-93 °C; Mol. For: $C_{18}H_{13}Cl_2N_3O_2S$; Mol. Wt: 406.28; IR (cm^{-1}): 1049 (C=S str); 840 (Ar C-Cl str); 2235 (C=N str); 3330 (OH str); 3114.4 (NH str); 2817.13 (CH str); 1394.58 (C-N str); 3121.18 (C=N str): ^{1}H NMR (400 MHz, DMSO-*d*$_6$): δ (ppm) 11.62 (s, 1H, OH), 10.81 (s, 1H, NH), 7.14-8.83 (m, 4H, Ar 1H), 6.13-6.95 (m, 3H, Ar 2H), 5.84 (s, 1H, N=CH), 2.82 (s, 3H, CH_3); ^{13}C NMR (100 MHz, DMSO-*d*$_6$): δ (ppm) 21.10 (CH_3), 108.40, 113.67, 126.73, 128.62 (Ar-C1), 135.11 (Ar CCl),

135.53 (Ar C-Cl), 159.96 (Ali C=N), 174.90 (C=S), 154.93 (Ar C=N), 156.72 (C-O), 136.49 (Ar C-CH3), 161.98 (C-OH), 131.72, 133.40, 98.36, 116.05, 129.16 (Ar-C2): MASS- 415.2001 (M+1), 405.01 ($C_{18}H_{13}Cl_2N_3O_2S$ m/z), 212.94 ($C_8H_4Cl_2NS$ m/z-3), 245.27 ($C_{11}H_9N_3O_2S_2$ m/z-2), 187.57 ($C_8H_4Cl_2NS$ m/z), 160.05 ($C_{10}H_8O_2$ m/z).

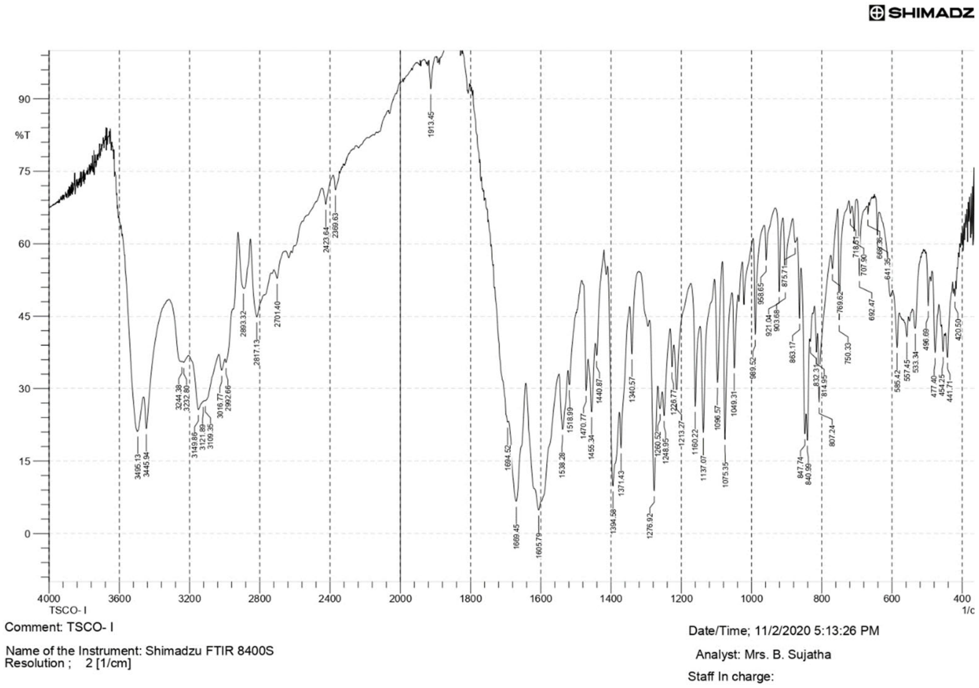

Figure S2. IR Spectra of **7a**

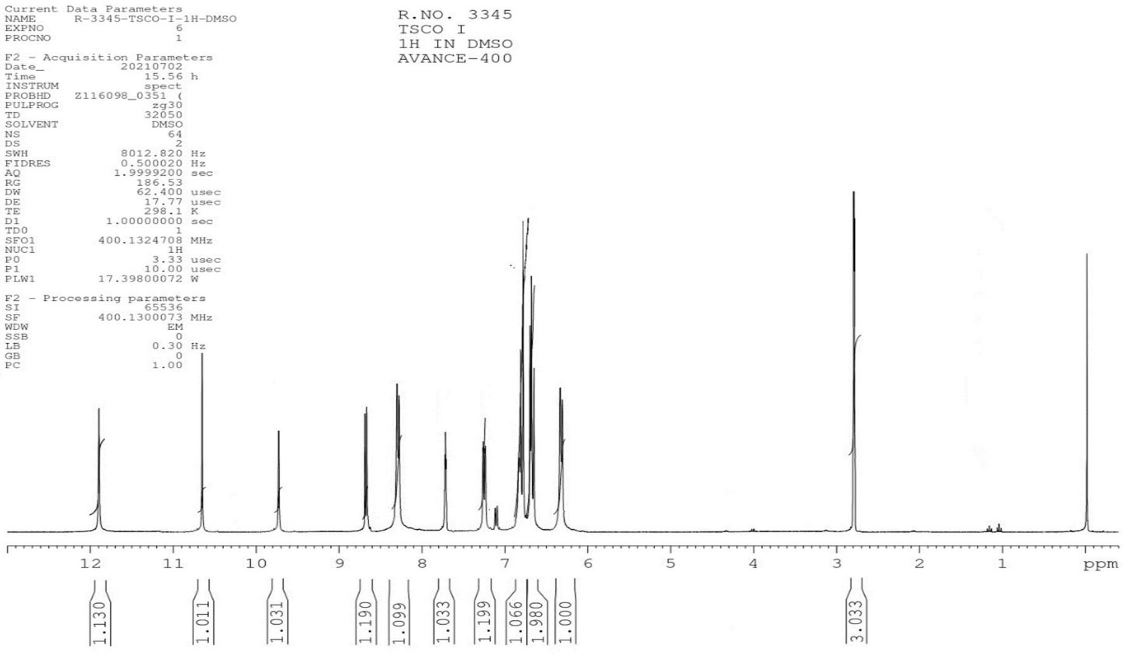

Figure S3. ^{1}H NMR Spectra of **7a**

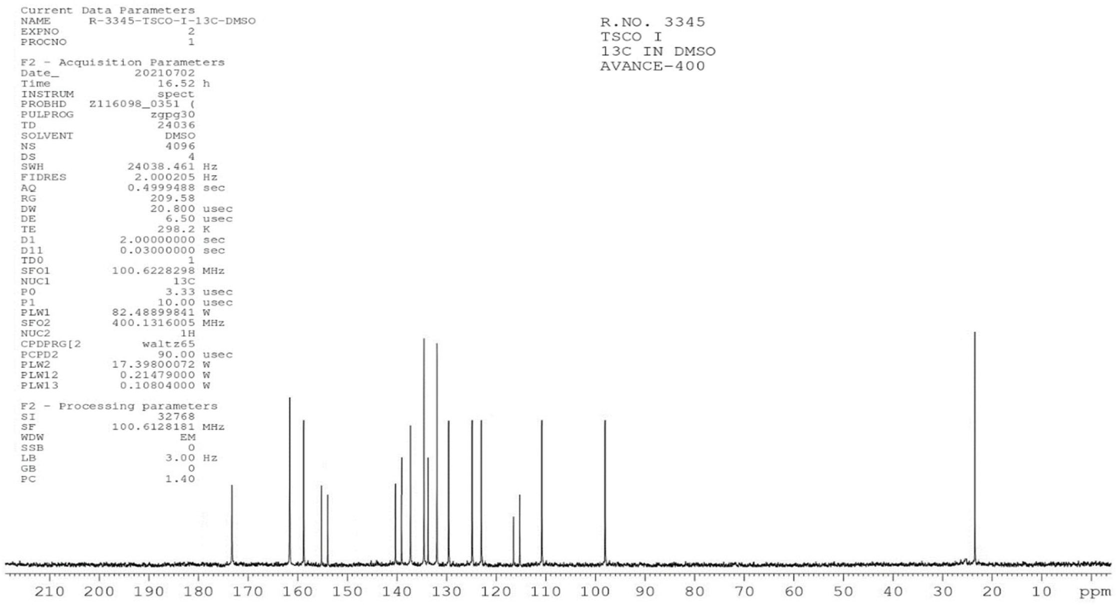

Figure S4. ^{13}C NMR Spectra of 7a

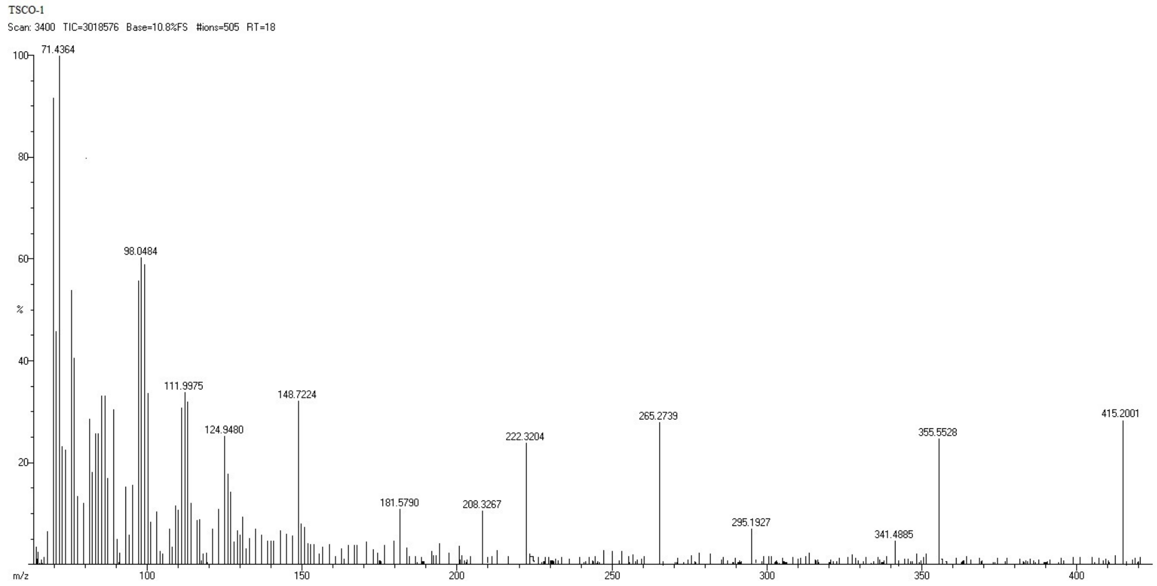

Figure S5. MASS Spectra of **7a**

2-((*E*)-7-hydroxy-4-methyl-2*H*-chromen-2-ylidene)-*N*-((*E*)-2-nitro benzylidene)hydrazine-1-carbothioamide (7f)

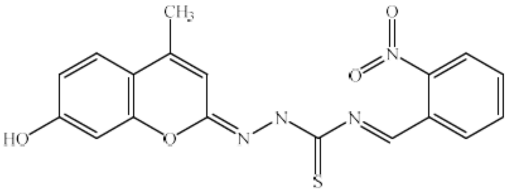

Yellow solid; Rf = 0.88, yield 73%. M.P: 96-98 °C; Mol. For: $C_{18}H_{14}N_4O_4S$; Mol. Wt-382.39. FTIR (KBr, cm-1): 1049 (C=S str); 2235 (C-N str); 3330 (OH str); 3114.4 (N-H stretch); 3048 (C-H str); 1150 (C-N str), 1524.68 (N-O str): ^{1}H NMR (400 MHz, DMSO-*d*$_6$): δ (ppm) 13.74 (s, 1H, OH), 10.85 (s, 1H, NH), 7.36-8.15 (m, 4H, Ar H1), 6.84-7.26 (m, 3H, Ar H2), 9.41 (s, 1H, N=CH), 2.73 (s, 3H, CH_3); ^{13}C NMR (100 MHz, DMSO-*d*$_6$): δ (ppm) 22.10 (CH3), 98.36, 113.67, 115.88, 117.41 (Ar-C2), 135.53 (Ar CNO2), 156.71 (Ali C=N), 178.90 (C=S), 154.94 (Ar C=N), 153.67 (C-O), 136.49 (Ar CCH_3), 161.98 (C-OH), 126.73, 128.19, 128.82, 129.71, 130.91, 131.84 (Ar-C1): MASS-382.3898 (M), 336.08 ($C_{18}H_{14}N_3O_2S$ m/z), 247.04 ($C_{11}H_9N_3O_2S_2$ m/z), 135.03 ($C_7H_5NO_2$ m/z).

2-((*E*)-7-hydroxy-4-methyl-2*H*-chromen-2-ylidene)-*N*-((*E*)-4-methoxybenzylidene) hydrazine-1-carbothioamide (7i)

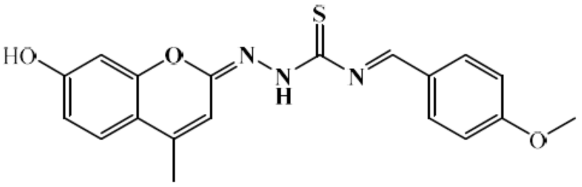

Solid with a light brown hue; Rf value of 0.24, yielding 75%. M.P: 116-118 °C; Mol. For: $C_{19}H_{17}N_3O_3S$; Mol. Wt-367.42. FTIR (KBr, cm^{-1}): 1100.43 (C=S str); 2361.91 (C-N str); 3493.20 (OH str); 3114.4 (N-H str); 3144.07 (C-H str); 1100.43 (C-N str), 2881.75 (-OCH_3 str): ^{1}H NMR (400 MHz, DMSO-*d*$_6$): δ (ppm) 13.35 (s, 1H, OH), 10.85 (s, 1H, NH), 6.38-6.97 (m, 4H, Ar H1), 7.55-7.63 (m, 3H, Ar H2), 8.20 (s, 1H, N=CH), 2.63 (s, 3H, CH_3), 3.76 (s, 3H, OCH_3); ^{13}C NMR (100 MHz, DMSO-*d*$_6$): δ (ppm) 21.10 (CH3), 55.28 (O-CH_3), 98.36, 108.40, 113.67, 114.74, 115.30 (Ar-C2), 163.12 (Ali C=N), 174.94 (C=S), 156.02 (Ar C=N), 163.15(C-O), 136.49 (Ar C-CH_3), 161.98 (C-OH), 126.73, 131.34, 134.70 (Ar-C1). MASS-367.4245 (M+1), 353.59 ($C_{18}H_{14}N_3O_3S$ *m*/*z*+1).

***N*-((*E*)-4-bromobenzylidene)-2-((*E*)-7-hydroxy-4-methyl-2*H*-chromen-2-ylidene)hydrazine-1-carbothioamide) (7m)**

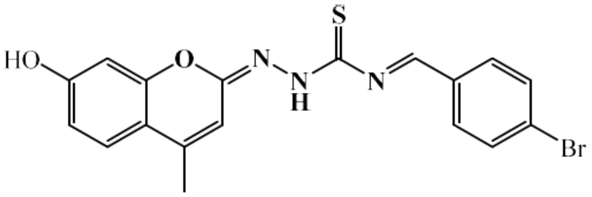

Brown solid; Rf = 0.70, yield 90%. M.P: 124-126 °C; Mol. For: $C_{18}H_{14}BrN_3O_2S$; Mol.Wt-416.29. FTIR (KBr, cm^{-1}): 1075 (C=S str); 3442.09 (C-N str); 3330 (OH str); 3496.09 (N-H str); 3068.85 (C-H str); 1160.22 (C-N str), 1137.07 (Ar-Br str): ^1H NMR (400 MHz, DMSO-d_6): δ (ppm) 13.33 (s, 1H, OH), 10.85 (s, 1H, NH), 6.38-7.57 (m, 4H, Ar H1), 7.63-8.13 (m, 4H, Ar H2), 9.41 (s, 1H, N=CH), 2.73 (s, 3H, CH_3); ^{13}C NMR (100 MHz, DMSO-d_6): δ (ppm) 21.10 (CH_3), 98.36, 108.40, 113.67, 115.88, 124.41 (Ar-C2), 135.53 (Ar C-NO2), 156.71 (Ali C=N), 174.90 (C=S), 154.94 (Ar C=N), 153.67 (C-O), 136.49 (Ar C-CH_3), 161.98 (C-OH), 126.00; 126.73; 130.10; 132.10; 134.70; 136.38 (Ar-C1): MASS-416.2942 (M+1), 351.10 ($C_{19}H_{17}N_3O_2S$ m/z).

2-((*E*)-4-hydroxy-2*H*-chromen-2-ylidene)-*N*-((*E*)-3,4,5-trihydroxy benzylidene) hydrazine-1 carbothioamide) (7n)

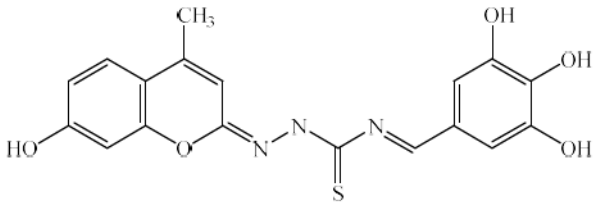

White solid; Rf = 0.67, yield = 81%. M.P: 126-128 °C; Mol. For: $C_{18}H_{15}N_3O_5S$; Mol. Wt-385.39. FTIR (KBr, cm^{-1}): 1075 (C=S str); 3442, 3446, 3498 (Ar OH str); 2367 (C=N str); 3330 (OH str); 3112.5 (NH str); 2818.09 (CH str); 1394.58 (C-N); 3112.25 (C=N str): ^1H NMR (400 MHz, DMSO-d_6): δ (ppm) 14.58 (s, 1H, OH), 10.85 (s, 1H, NH), 6.59-6.84 (m, 4H, Ar H1), 6.85 (m, 2H, Ar H2), 8.22 (s, 1H, N=CH), 2.73 (s, 3H, CH_3), 5.02 (s, 3H, Ar-OH): ^{13}C NMR (100 MHz, DMSO-d_6): δ (ppm) 21.10 (CH_3), 98.36, 108.40, 110.68, 113.67 (Ar-C2), 137.30 (Ar C-OH), 146.57 (Ar C-OH), 161.98 (Ali C=N), 175.01 (C=S), 156.15 (Ar C=N), 154.91 (C-O), 136.49 (Ar C-CH_3), 163.63 (C-OH), 115.10 (Ar-C), 126.73 (Ar-C), 132.06 (Ar-C), 135.91 (Ar-C) (Ar-C1): MASS- 385.3900 (M+1). 225.02 ($C_8H_7N_3O_3S_2$), 194.6766 ($C_8H_6NO_3S$ m/z-2), 140.7000 ($C_7H_6O_3$).

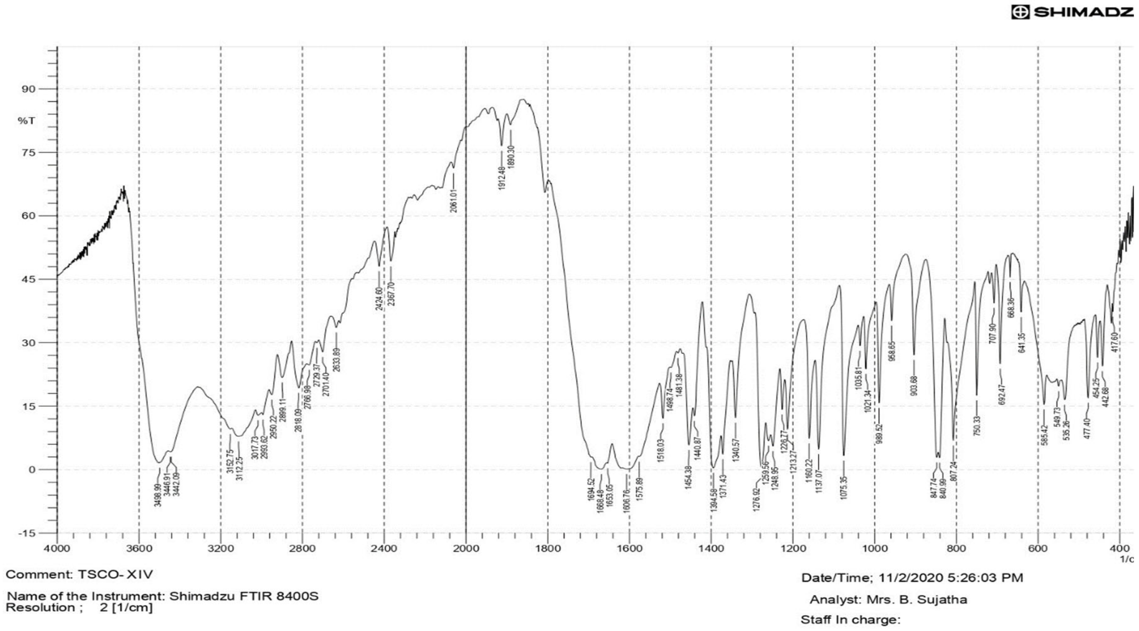

Figure S6. IR Spectra of **7n**

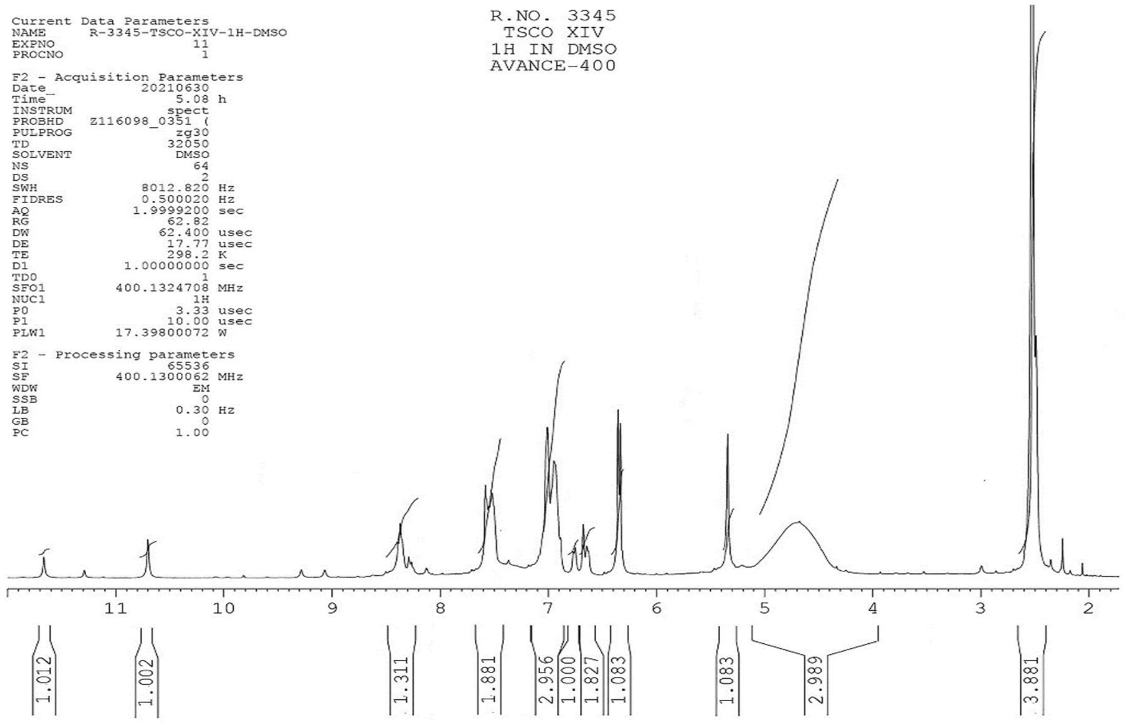

Figure S7. ^{1}H NMR Spectra of **7n**

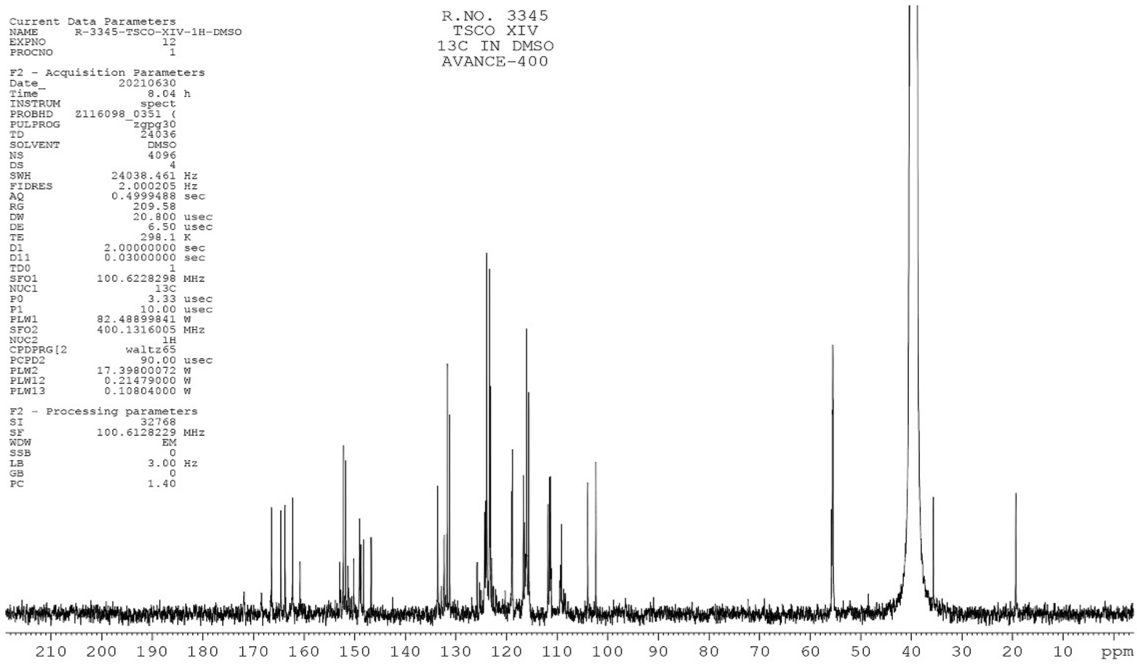

Figure S8. ^{13}C NMR Spectra of 7n

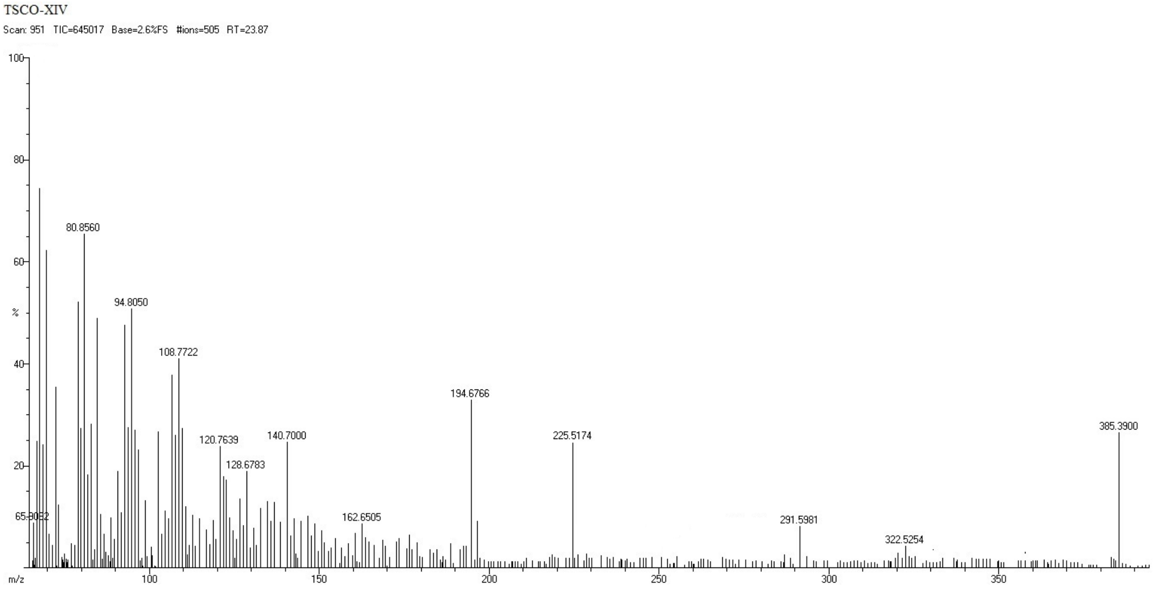

Figure S9. MASS Spectra of **7n**

Scheme-II

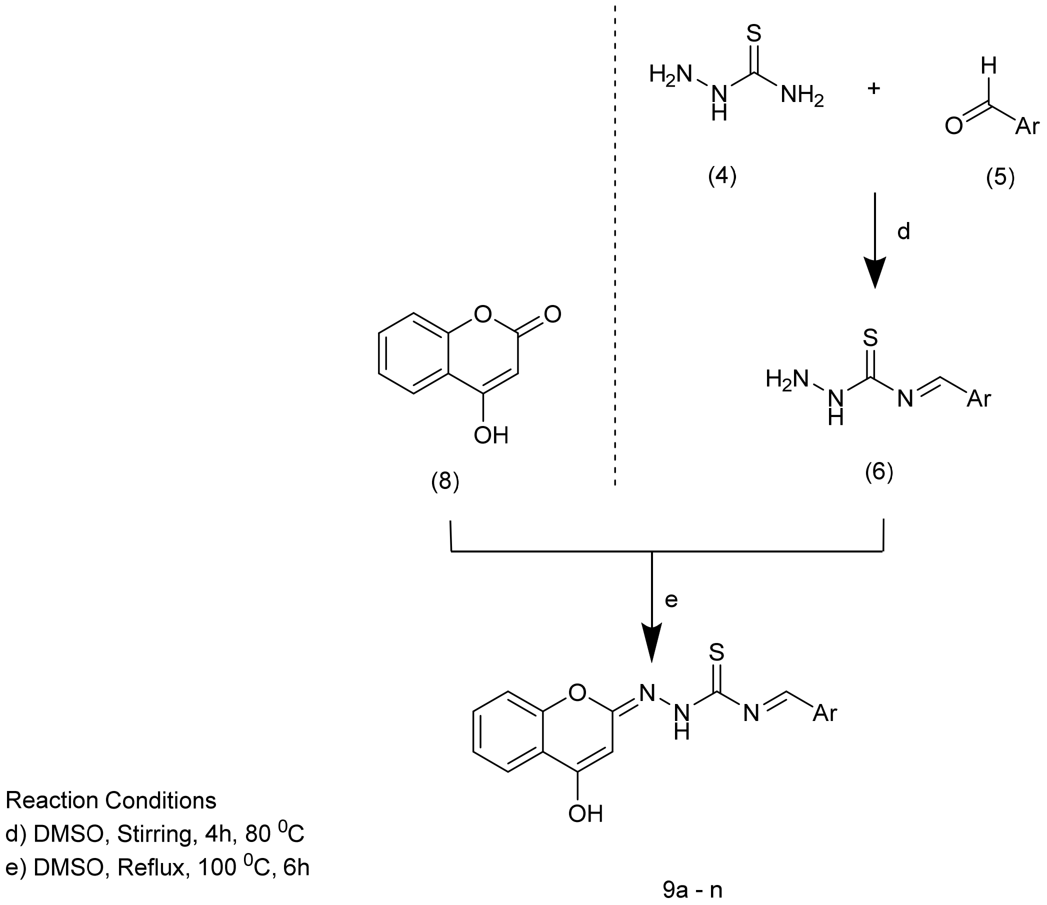

Figure S10. Scheme II. Synthesis of *N*-((*E*)-benzylidene)-2-((*E*)-7-hydroxy-4-methyl-2*H*-chromen-2-ylidene) hydrazine-1-carbothioamide

***N*-((*E*)-2,4-dichlorobenzylidene)-2-((*E*)-4-hydroxy-2*H*-chromen-2-ylidene) hydrazine-1-carbothioamide) (9a)**

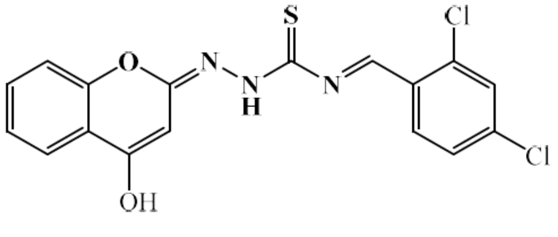

Solid with a yellow hue; Rf value of 0.52, yielding 78%. M.P: 113-115 °C; Mol. For: $C_{17}H_{11}Cl_2N_3O_2S$; Mol. Wt-392.25. FTIR (KBr, cm^{-1}): 1049 (C=S str); 840 (Ar C-Cl str); 2235 (C=N str); 3330 (OH str); 3114.4 (NH str); 2817.13 (CH str); 1394.58 (C-N) str; 3121.18 (C=N str): ^{1}H NMR (400 MHz, DMSO-d_6): δ (ppm) 13.25 (s, 1H, OH), 10.65 (s, 1H, NH), 6.60-7.48 (m, 5H, Ar H1), 7.69-8.23 (m, 3H, Ar H2), 8.39 (s, 1H, N=CH): ^{13}C NMR

(100 MHz, DMSO-d_6): δ (ppm) 101.29, 113.62, 116.51, 123.20, 123.40 (Ar-C2), 135.11 (Ar CCl), 135.53 (Ar C-Cl), 155.81 (Ali C=N), 174.89 (C=S), 158.63 (Ar C=N), 153.18 (C-O), 158.98 (C-OH), 131.73, 133.40, 133.76 (Ar-C1): MASS- 392.99 ($C_{17}H_{11}Cl_2N_3O_2S$ M+).

***N*-((*E*)-4-chlorobenzylidene)-2-((*E*)-4-hydroxy-2*H*-chromen-2-ylidene) hydrazine-1-carbothioamide) (9b)**

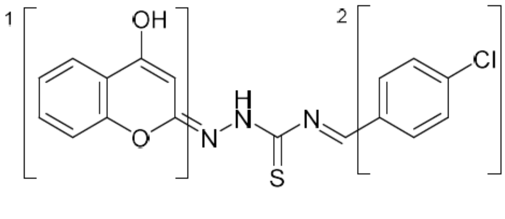

Solid with an ash color; Rf = 0.46, yield 72%. M.P: 126-128 °C; Mol. For: $C_{17}H_{12}ClN_3O_2S$; Mol. Wt-357.81. FTIR (KBr, cm-1): 1075.35 (C=S str); 840.99 (Ar C-Cl str); 2363.84 (C=N str); 3496.09 (OH str); 3096.2 (NH str); 2817.13 (CH str); 1394.58 (C-N str); 3152.75 (C=N str): ^{1}H NMR (400 MHz, DMSO-d_6): δ (ppm) 13.63 (s, 1H, OH), 10.65 (s, 1H, NH), 6.63-7.96 (m, 5H, Ar H1), 7.40 -7.71 (m, 4H, Ar-H2), 8.17 (s, 1H, N=CH): ^{13}C NMR (100 MHz, DMSO-d_6): δ (ppm) 101.12, 113.61, 116.51, 123.20, 123.40 (Ar- C2), 137.40 (Ar C-Cl), 159.34 (Ali C=N), 174.93 (C=S), 155.80 (Ar C=N), 153.19 (CO), 162.56(C-OH), 129.39, 129.44, 133.76, 134.70(Ar-C1): MASS- 371.5423 (M+1), 357.03 ($C_{17}H_{12}ClN_3O_2S$), 337.09 ($C_{18}H_{15}N_3O_2S$), 211.00($C_8H_6ClN_3S_2$), 181.98 (C_8H_5ClNS), 124.01($C_7H_5Cl_2$).

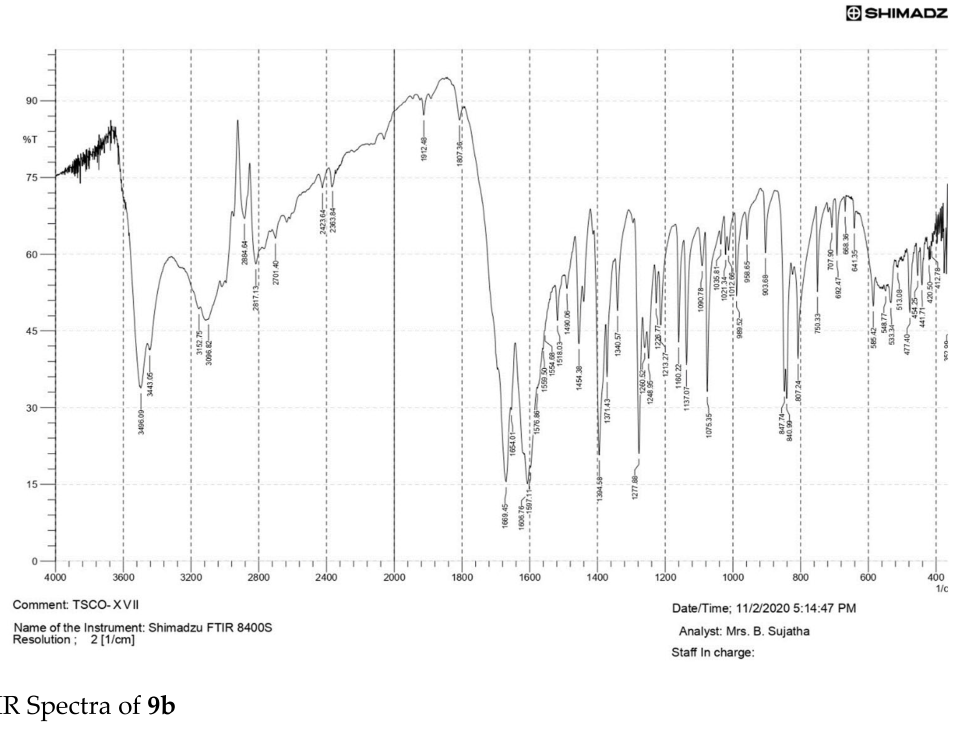

Figure S11. IR Spectra of **9b**

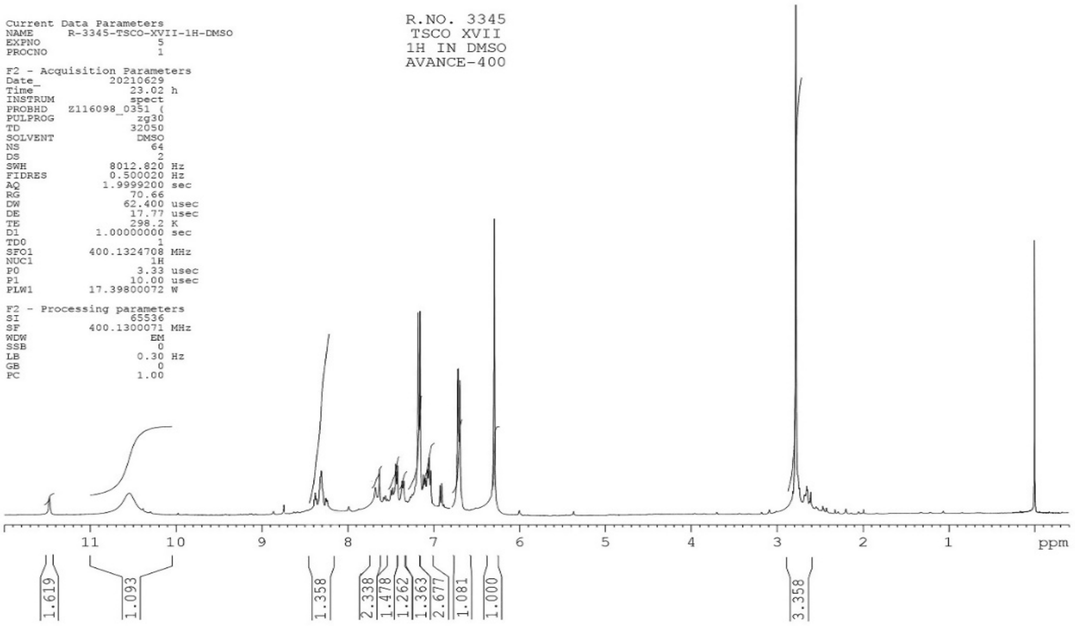

Figure S12. ^{1}H Spectra of **9b**

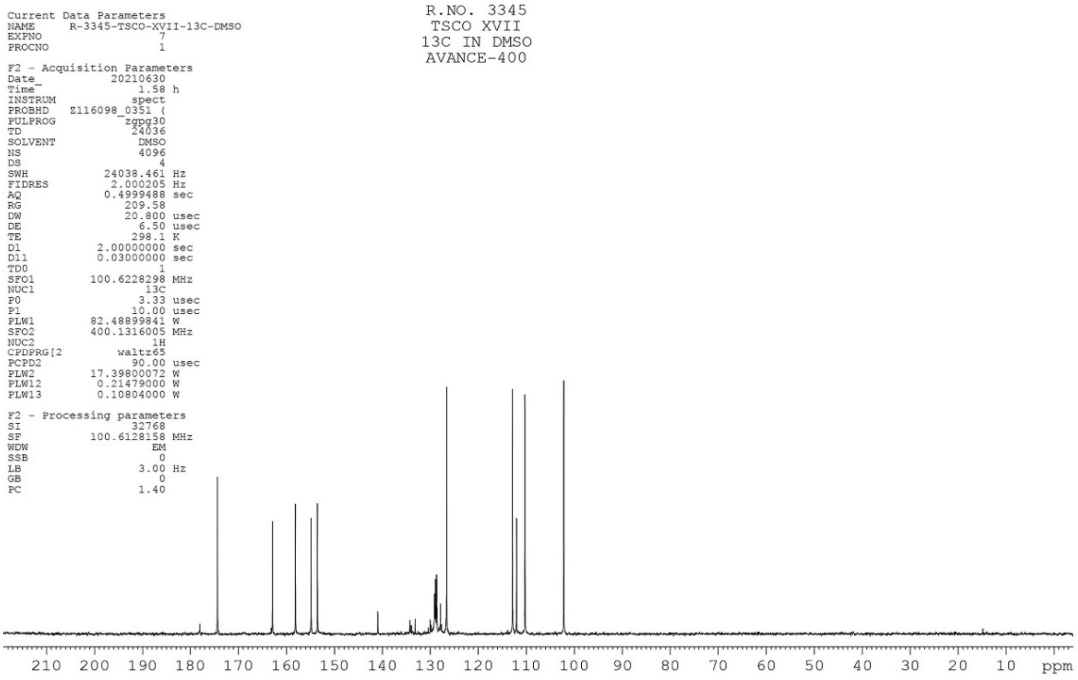

Figure S13. ^{13}C NMR Spectra of **9b**

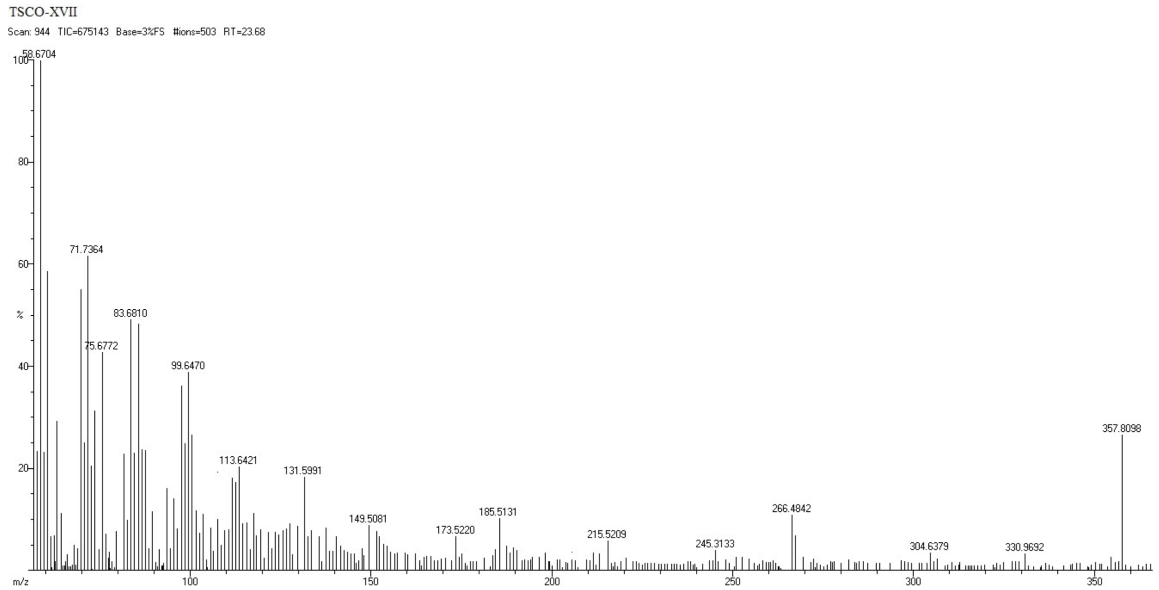

Figure S14. MASS Spectra of 9b

2-((*E*)-4-hydroxy-2*H*-chromen-2-ylidene)-*N*-((*E*)-naphthalen-1-ylmethylene) hydrazine-1-carbothioamide) (9c)

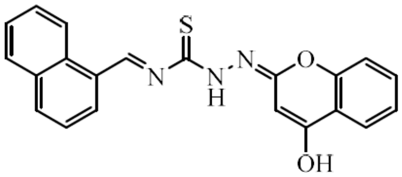

Solid with a dark brown color; Rf = 0.25, yield 79%. M.P: 147-149 °C; Mol. For: $C_{21}H_{15}N_3O_2S$; M. Wt-373.43. FTIR (KBr, cm^{-1}): 1075.35 (C=S str); 2359.98 (C=N str); 3493.20 (OH str); 3096.82 (NH str); 2817.13 (CH str); 1394.58 (C-N str); 3151.79 (C=N str) ; ^{1}H NMR (400 MHz, DMSO-d_6): δ (ppm) 13.56 (s, 1H, OH), 10.65 (s, 1H, NH), 6.74-7.69 (m, 7H, Ar H1), 7.71-8.42 (m, 5H, Ar-H), 8.76 (s, 1H, N=CH): ^{13}C NMR (100 MHz, DMSO-d_6): δ (ppm) 100.90, 113.47, 116.51, 123.20, 123.40, 124.02, 124.68, 125.76, 126.56 (Ar-C2), 155.56 (Ali C=N), 174.95 (C=S), 153.47 (Ar C=N), 152.77 (C-O), 159.28(C-OH), 127.39, 127.40, 128.46, 129.02, 130.38, 133.20, 133.76(Ar-C1): MASS- 405. 2691 (M+1), 238.03 ($C_{10}H_7N_3O_2S_2$ m/z+5), 208.41($C_{12}H_8NS$ m/z+5), 200.19 ($C_{12}H_8NS$ m/z+2), 171.57 ($C_9H_7N_2O_2$ m/z-4), 148.72 ($C_9H_6O_2$ m/z+2).

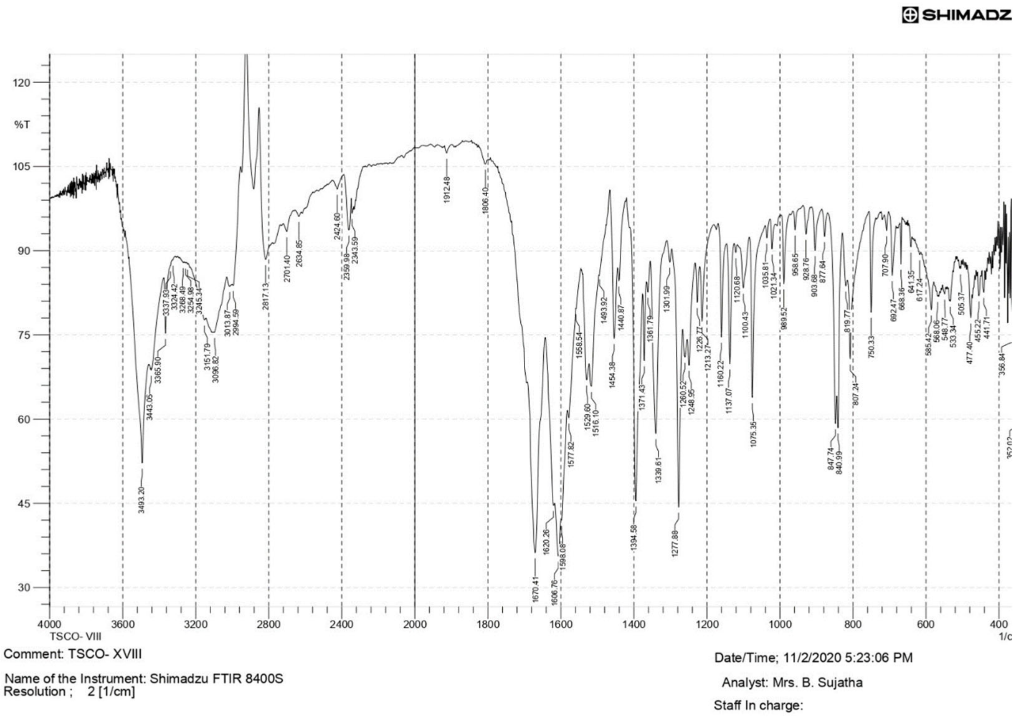

Figure S15. IR Spectra of **9c**

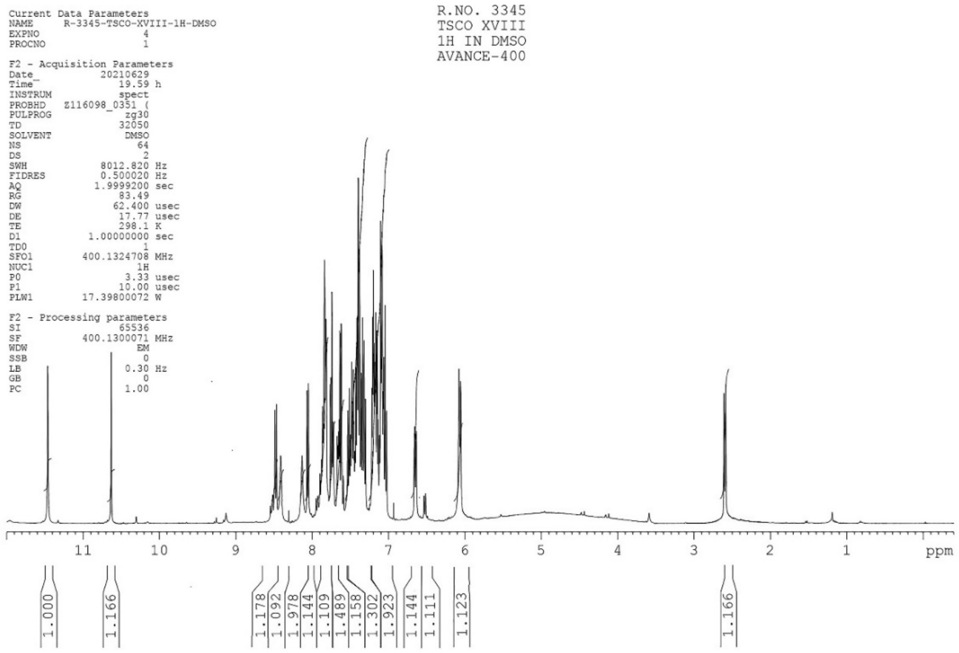

Figure S16. ^{1}H NMR Spectra of **9c**

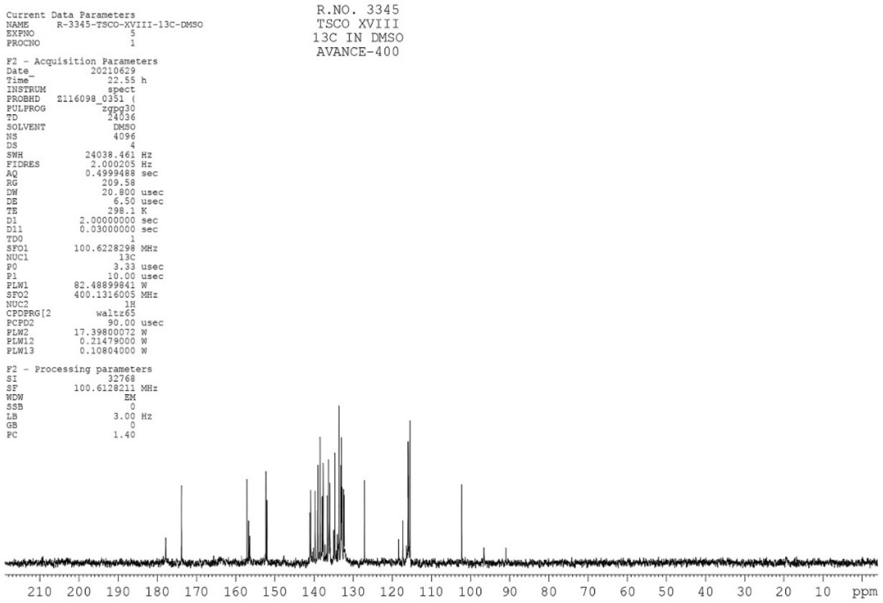

Figure S17. ^{13}C NMR Spectra of **9c**

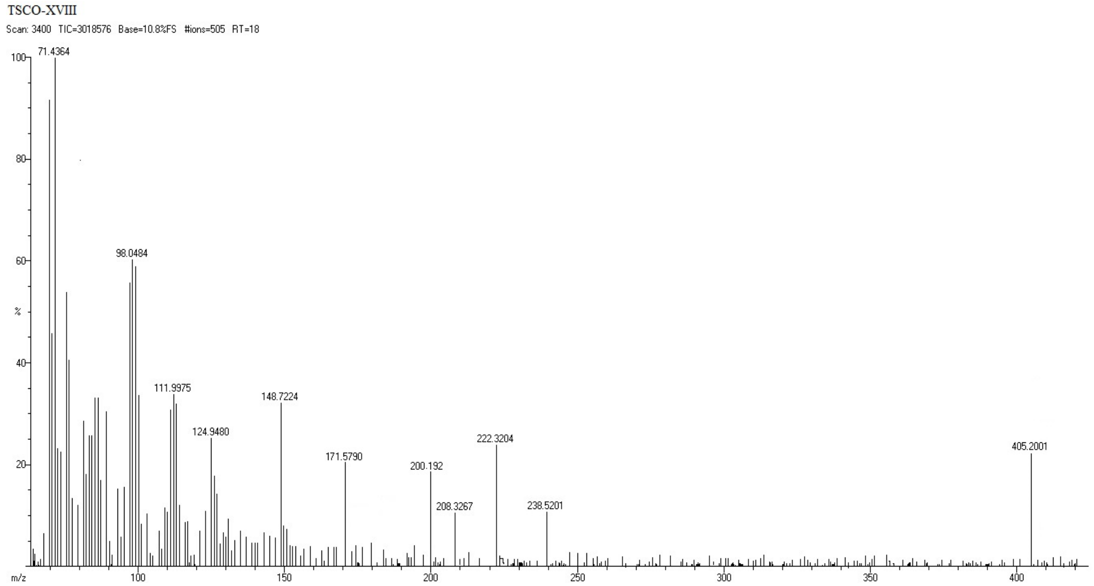

Figure S18. MASS Spectra of **9c**

2-((*E*)-4-hydroxy-2*H*-chromen-2-ylidene)-*N*-((*E*)-4-methyl benzylidene) hydrazine-1-carbothioamide) (9l)

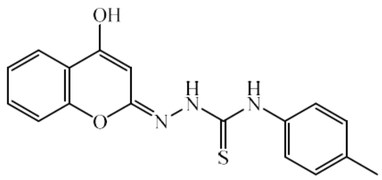

Solid with a pale brown color; Rf value of 0.61, yielding 81%. M.P: 107-109 °C; Mol. For: $C_{18}H_{15}N_3O_2S$; Mol. Wt-337.40; FTIR (KBr, cm^{-1}): 1075.35 (C=S str); 2361.91 (C=N str); 3444.02 (OH str); 3148.90 (NH str); 2813.27 (CH str); 1398.44 (C-N); 3267.52 (C=N str): ^{1}H NMR (400 MHz, DMSO-d_6): δ (ppm) 12.74 (s, 1H, OH), 9.70 (s, 1H, NH), 6.69-7.94 (m, 5H, Ar H1), 7.14 (m, 2H, Ar-H), 7.55 (m, 2H, Ar-H): ^{13}C NMR (100 MHz, DMSO-d_6): δ (ppm) 20.68 (CH_3), 100.98, 113.61, 116.51, 123.20, 123.40, 125.60, 125.61 (Ar-C2), 155.80 (Ali C=N), 181.40 (C=S), 153.47 (Ar C=N), 152.77 (C-O), 159.34 (C-OH), 130.70, 130.71, 133.30, 133.76, 137.77, 154.64 (Ar-C1): MASS- 337.3999 (M+1), 321.09 ($C_{17}H_{15}N_3O_2S$ m/z-4), 179.05 ($C_8H_9N_3S_2$), 152.04 (C_8H_8NS m/z+2), 106.1498 (C_7H_8N).

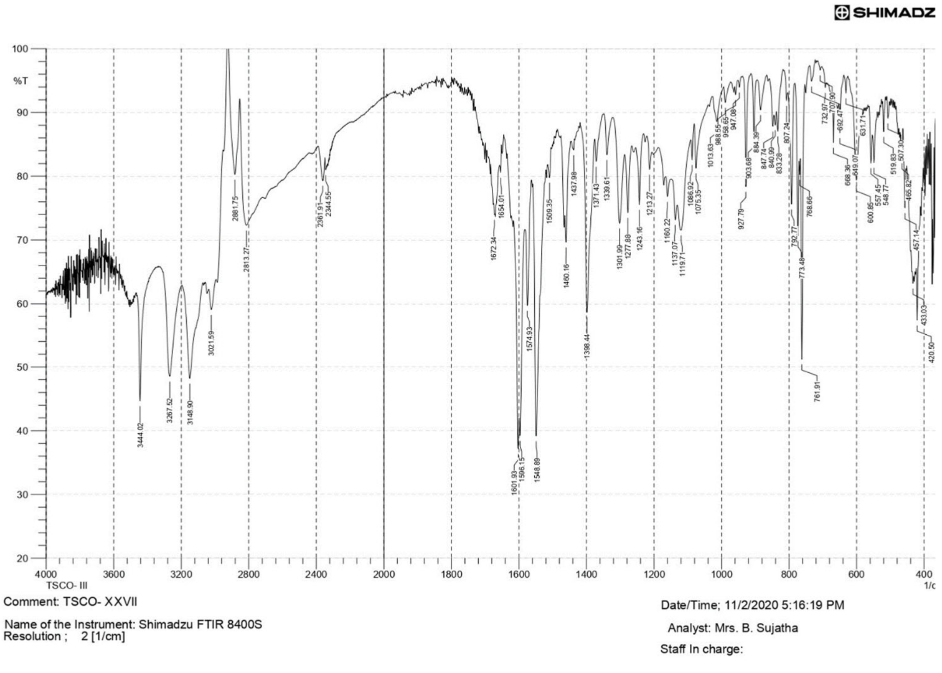

Figure S19. IR Spectra of **9l**

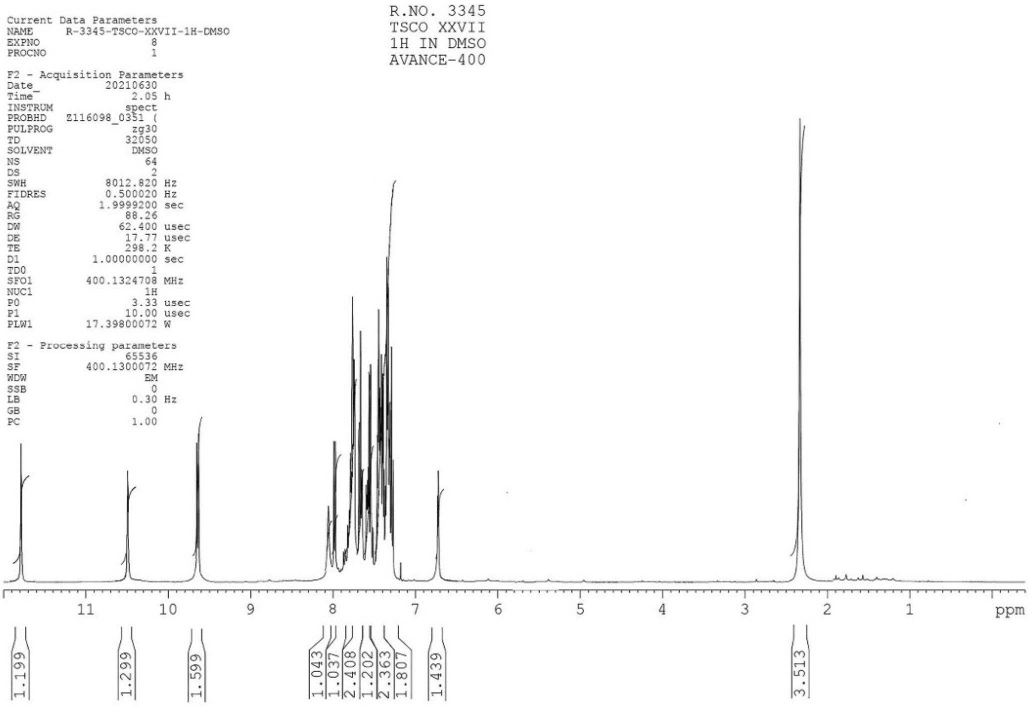

Figure S20. ^{1}H NMR Spectra of **9l**

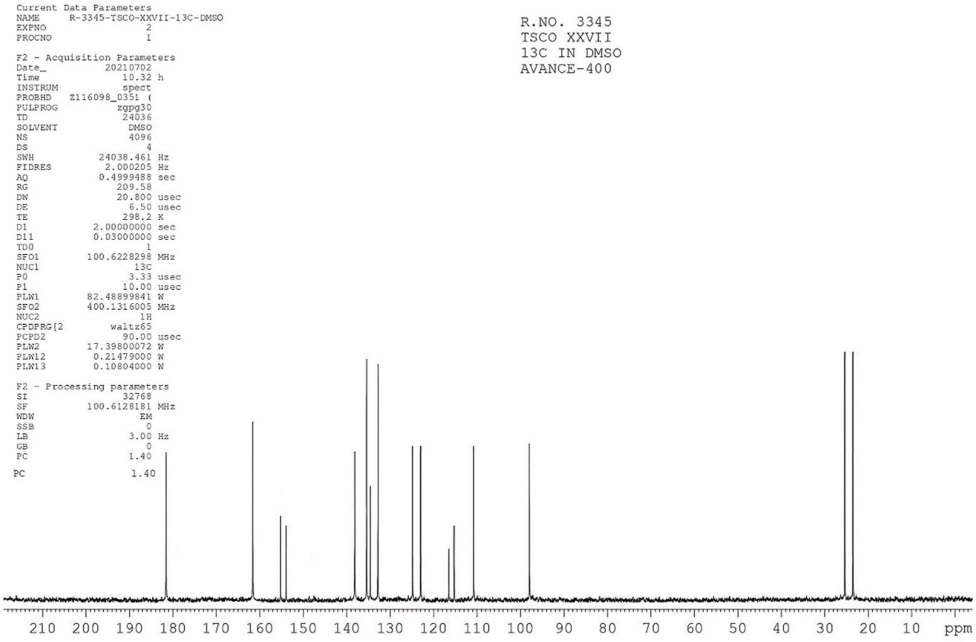

Figure S21. ^{13}C NMR Spectra of **9l**

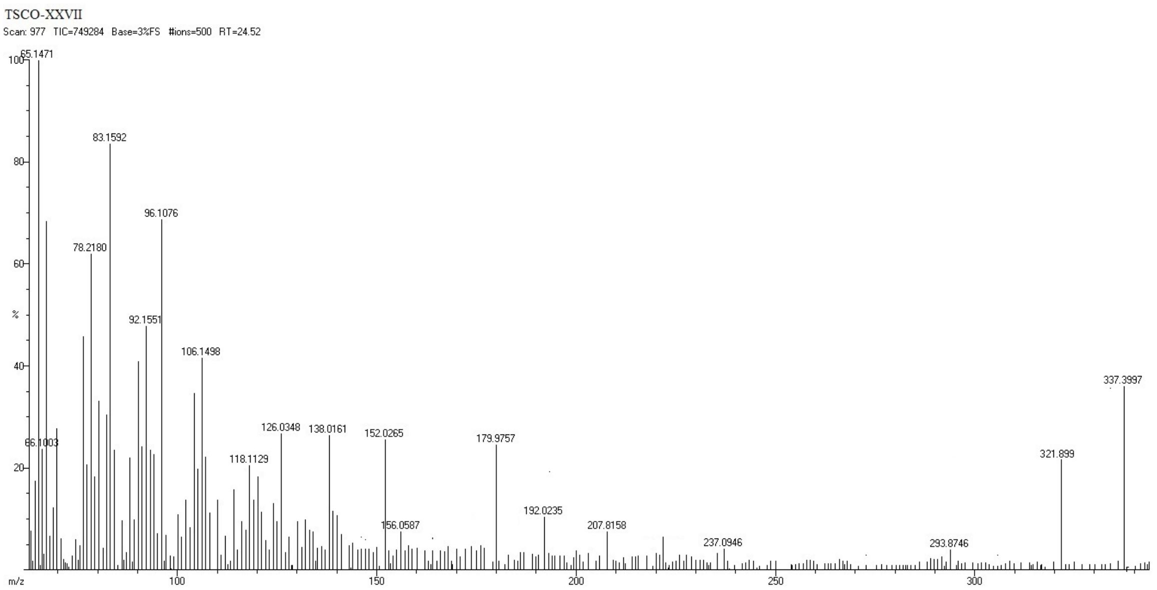

Figure S22. MASS Spectra of **9l**

2-((*E*)-4-hydroxy-2*H*-chromen-2-ylidene)-*N*-((*E*)-3,4,5-trihydroxybenzylidene) hydrazine-1-carbothioamide (9n)

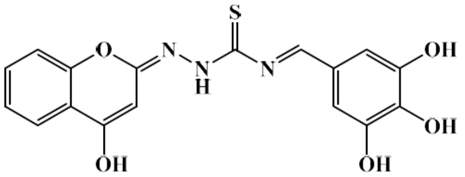

Yash color solid; Rf = 0.61, yield 81%. M.P: 107-109 °C; Mol. Formula: $C_{17}H_{13}N_3O_5S$; M. Wt- 371.37; FTIR (KBr, cm^{-1}): 1075.35 (C=S str); 2362.88 (C=N str); 3495.13 (OH str); 3148.90 (NH str), 2825.81 (CH str); 1393.62 (C-N): ^{1}H NMR (400 MHz, DMSO-d_6): δ (ppm) 14.87 (s, 1H, OH), 10.65 (s, 1H, NH), 5.02 (s, 2H, OH); 5.02 (s, 1H, OH); 6.67 (s, 1H, OH), 7.11-8.00 (m, 5H, Ar H1), 6.74 (d, 2H, Ar-H), 8.30 (s, 1H, N=CH): ^{13}C NMR (100 MHz, DMSO-d_6): δ (ppm) 100.34, 110.75, 113.60, 116.51, 123.20, 123.40, 132.31(Ar-C2), 137.30 (ArC-OH) 162.29 (Ali C=N), 175 (C=S), 153.16 (Ar C=N), 155.24 (C-O), 160.08 (C-OH), 133.76 (Ar-C2): MASS-371(m/z+1), 371.06($C_{17}H_{13}N_3O_5S$ m/z), 225.02($C_8H_7N_3O_3S_2$ m/z), 197.25 ($C_8H_6NO_3S$ m/z+1), 141.34($C_7H_6O_3$ m/z+3).

Table S1. *In vitro* cytotoxicity on MDA MB 231 cell lines images

Compound Code	Microscopic Image
**9b (**14.49μM)	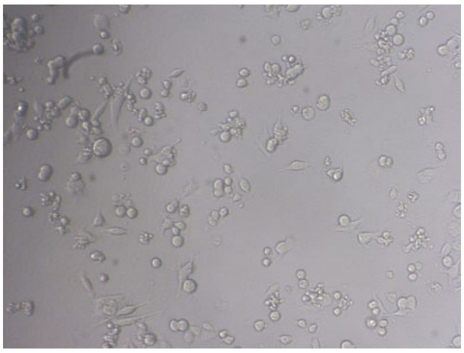
**9l** (35.08μM)	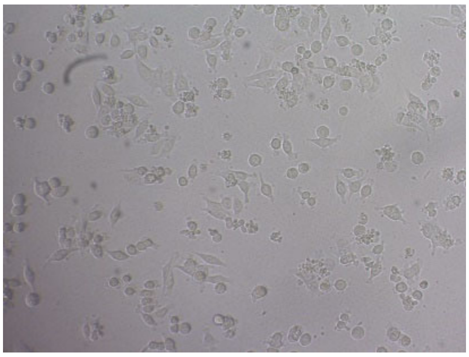
**9m** (42.12 μM)	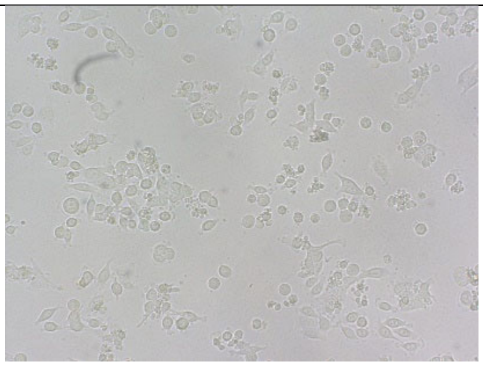

**9n** (47.72 μM)	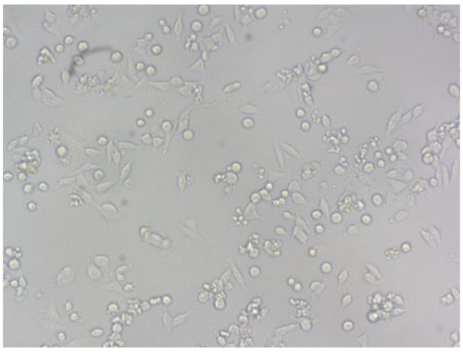
**7h** (80.30μM)	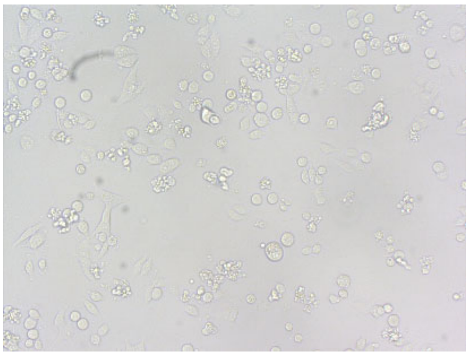
**7b** (70.46 μM)	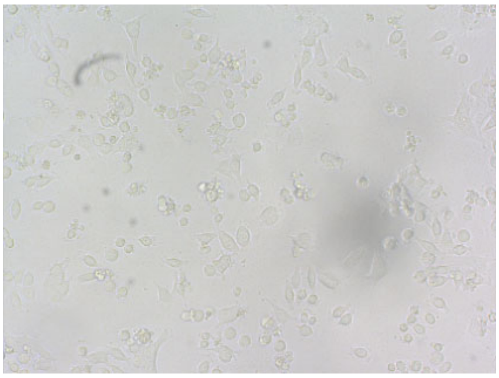

**9c** (61.48μM)	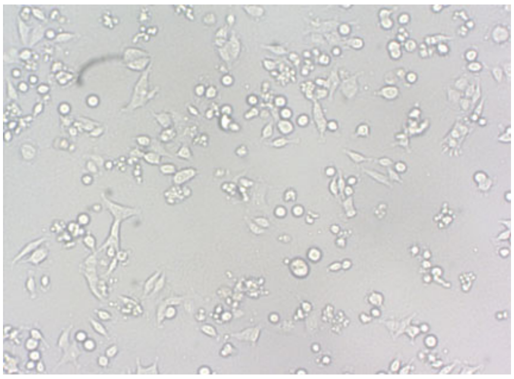
**7m** (90.49μM**)**	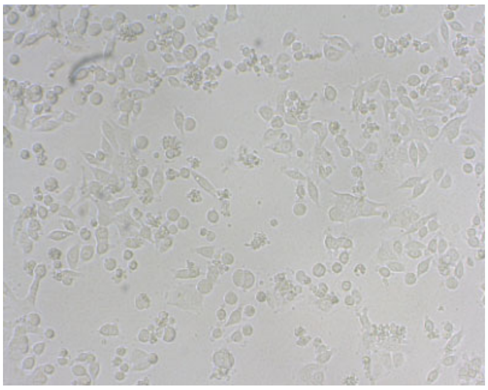
**7o** (136.98 μM)	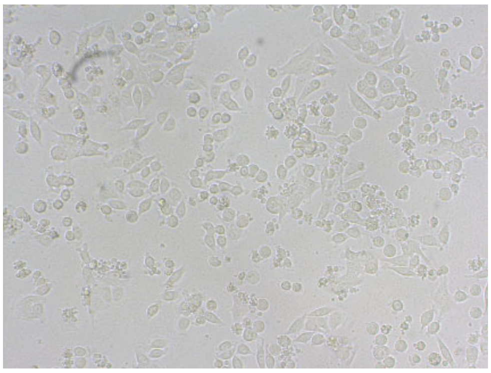

Gene Expression Studies

Fluorescence vs Cycle for Cyclin D1

Table S2. Real-Time PCR Raw Data for Cycling A. Green Ct Values for Cyclin D1

No.	Color	Name	Type	Ct
1		GAPDH C	Unknown	32.93
2		GAPDH C	Unknown	33.93
3		GAPDH C	Unknown	33.28
4		CYCLIND1 C	Unknown	27.47
5		CYCLIND1 C	Unknown	28.30
6		CYCLIND1 C	Unknown	27.70
7		GAPDH	Unknown	34.88
8		GAPDH	Unknown	35.99
9		GAPDH	Unknown	34.09
10		1S	Unknown	31.72
11		1S	Unknown	32.15
12		1S	Unknown	31.00
13		2S	Unknown	28.96
14		2S	Unknown	28.83
15		2S	Unknown	28.77
16		3S	Unknown	31.40
17		3S	Unknown	31.58
18		3S	Unknown	31.39
19		4S	Unknown	27.63
20		4S	Unknown	27.13
21		4S	Unknown	27.38

Table S3. Real-Time PCR Raw Data for Cycling A. Green Ct Values for Bcl2

No.	Color	Name	Type	Ct
1		Bcl2 C	Unknown	29.11
2		Bcl2 C	Unknown	30.69
3		Bcl2 C	Unknown	30.60

4		GAPDH C	Unknown	30.65
5		GAPDH C	Unknown	31.12
6		GAPDH C	Unknown	31.86
7		GAPDH	Unknown	30.58
8		GAPDH	Unknown	31.77
9		GAPDH	Unknown	29.75
10		1S	Unknown	30.11
11		1S	Unknown	30.42
12		1S	Unknown	30.35
13		2S	Unknown	33.48
14		2S	Unknown	33.49
15		2S	Unknown	33.31
16		3S	Unknown	31.14
17		3S	Unknown	31.04
18		3S	Unknown	30.95
19		4S	Unknown	31.10
20		4S	Unknown	30.14
21		4S	Unknown	31.86
